# Novel Optical Imaging Probe for the Targeted Visualization of NLRP3 Inflammasomes in Living Retina

**DOI:** 10.1021/acs.jmedchem.5c00999

**Published:** 2025-08-04

**Authors:** MD Imam Uddin, Blake Dieckmann, Sarah Soliman

**Affiliations:** † Department of Ophthalmology and Visual Sciences, 12327 Vanderbilt University School of Medicine, Nashville, Tennessee 37232, United States; ‡ Department of Biomedical Engineering, Vanderbilt University School of Engineering, Nashville, Tennessee 37235, United States

## Abstract

Activation of the NLR-family pyrin domain-containing 3 (NLRP3) inflammasome has been associated with diabetic retinopathy progression. We have synthesized a highly sensitive molecular imaging probe, InflammaProbe-2, as an early detection diagnostic tool for the in vivo molecular imaging of NLRP3 inflammasomes in living diabetic retina. The ability of InflammaProbe-2 for the targeted visualization of the NLRP3 inflammasome was assessed using an enzyme-linked immunosorbent assay (ELISA) by comparing its ability to inhibit the NLRP3-mediated secretion of IL-1β. InflammaProbe-2 was able to visualize NLRP3 inflammasomes in ARPE-19 cells treated under hyperglycemia as well as inflammatory conditions. Furthermore, InflammaProbe-2-dependent in vivo and ex vivo imaging of the NLRP3 inflammasome was achieved via fluorescence enhancement in streptozotocin (STZ)-induced diabetic retinopathy. In addition, the toxicity of InflammaProbe-2 was assessed in vitro and in vivo studies. InflammaProbe-2 showed no significant changes in the a-wave and b-wave amplitudes of their electrical response to flashes of light.

## Graphical abstract

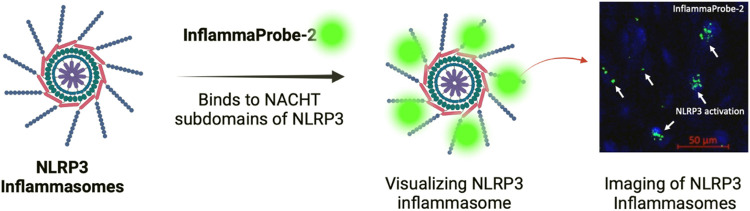

## Introduction

Diabetic retinopathy (DR) is a vision-threatening disease affecting a large number of working-age populations worldwide.^1,2^ The NLRP3 inflammasome is a multiprotein complex that amplifies inflammatory cytokines in the DR. NLRP3 inflammasome serves as a platform for caspase-1 activation in response to hyperglycemia,^3^ cellular damage,^4,5^ or infection.^6^ In diabetic retinopathy, NLRP3 inflammasome causes caspase-1-mediated programmed cell death (pyroptosis) in the retina.^7^ Active caspase-1 proteolyzes the biologically inert pro-IL-1β and pro-IL-18 cytokines into their bioactive inflammatory cytokines. In addition, caspase-1 proteolyzes Gasdermin D (GSDMD), resulting in pyroptotic cell death in diabetic retinopathy.^8^ Generally, NLRP3 inflammasome is composed of a sensor protein, caspase-1, and adapter proteins that assemble in response to pathogen- or danger-associated molecular patterns.^9^ NLRP3 inflammasome activation is regulated in two steps: (a) priming and (b) activation of the NLRP3 inflammasomes.^10,11^ Priming regulates NLRP3 and IL-1β mRNA production upon NF-κB activation.^12^ Although danger-associated molecular patterns (DAMPs) and pathogen-associated molecular patterns (PAMPs) are known to activate NLRP3 inflammasomes, the exact mechanism of NLRP3 inflammasome activation in DR is largely unknown. It is known that NLRP3 inflammasome activation can induce pyroptosis, leading to inflammatory cell death in DR.^13^ In addition, NLRP3 inflammasome directly contributes to DR pathology by regulating VEGF secretion.^14^ Pathologic VEGF is also strongly associated with NLRP3 inflammasome activation.^15^ Thus, imaging NLRP3 inflammasome activation could provide molecular details in the diabetic retina, predict the onset of DR and its progression, and monitor the response to anti-VEGF therapy.

The eye is optically accessible to several different types of imaging instrumentation, including optical coherence tomography (OCT) and confocal scanning laser ophthalmoscopy (cSLO) imaging systems. Fluorescence dyes such as fluorescein sodium and indocyanine green (ICG) have been used in the clinic for diagnostic applications in retinal vascular diseases.^16,17^ Although these dyes are currently used for retinal imaging to monitor circulation and vascular permeability, they can also be conjugated to molecularly targeted compounds to detect cellular subtypes and molecular expressions in vivo. For example, molecular biomarkers could be imaged using targeted imaging probes in the living retina.^18–27^ Although limited, essential progress has been made in developing protein- and mRNA-targeted contrast agents for retinal fluorescence imaging. The prospect of combining structural and functional imaging readouts obtained with fluorescein or ICG angiography with molecular imaging would provide powerful diagnostic information for the improved management of diabetic retinopathy.

Ophthalmic imaging techniques available in the clinic are not capable of imaging NLRP3 inflammasomes in the living retina. For example, the optical imaging method OCT is a high-resolution, noninvasive optical imaging method that is widely used to diagnose diabetic retinopathy, but this imaging method is not able to detect NLRP3 inflammasomes and distinguish inflammatory cells from normal cells. Similar imaging technique, adaptive optics scanning laser ophthalmoscopy (AO-SLO), was used to track the spatiotemporal dynamics of GFP^+^ microglia in a mouse model of photoreceptor damage,^28^ but this imaging method is not able to detect NLRP3 inflammasomes in activated cells. To this end, our newly designed chemical probe, InflammaProbe-2, provides a powerful technique to detect NLRP3 inflammasomes in the retina (Figure 1). Previously, we synthesized InflammaProbe-1 to detect NLRP3 inflammasome-associated macrophages in the subretinal space.^29^ InflammaProbe-1 can detect both activated and nonactivated NLRP3. We have further developed InflammaProbe-2 as a sensitive molecular imaging probe to detect only activated NLRP3 inflammasomes in diabetic retinopathy. Mechanistically, InflammaProbe-1 is a derivative of CY09, which possibly binds to the ATP-binding site of the NACHT domain,^30^ and thus InflammaProbe-1 could widely detect both NLRP3 and its activated inflammasomes. However, the new InflammaProbe-2, featuring a clinically relevant fluorophore conjugated to the NLRP3-targeted MCC950 moiety, presumably binds to the cavity between the NACHT subdomains of NLRP3,^31^ stabilizing the closed conformation of NLRP3 inflammasome, allowing imaging of the NLRP3 inflammasome activations only. We report here our results.

**Figure 1:**
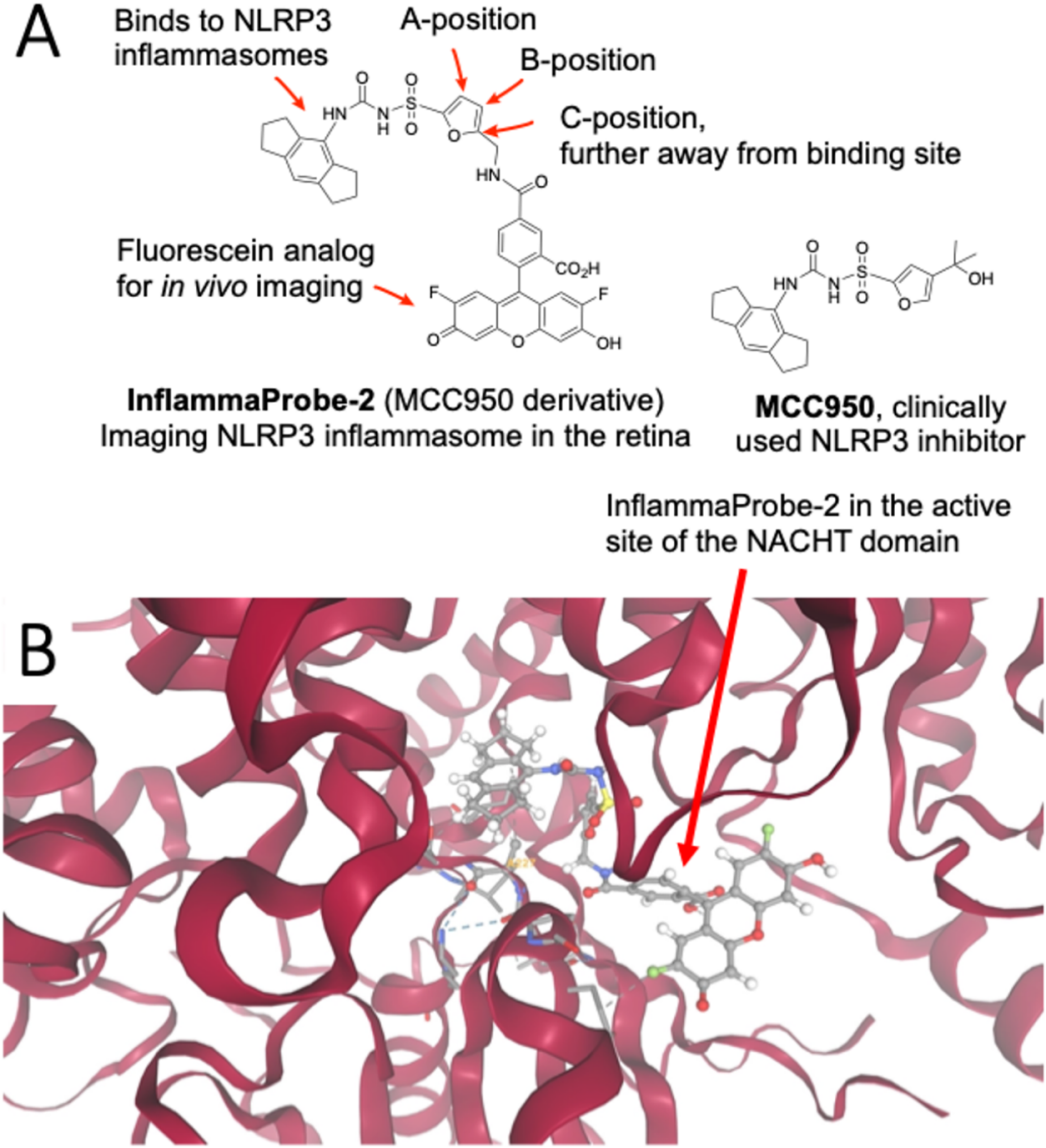
Chemical structure of InflammaProbe-2, a molecular imaging probe for the detection of NLRP3 inflammasome in the living retina. (A) InflammaProbe-2 was structurally designed by derivatizing the clinically used NLRP3 inhibitor, MCC950, to a clinically used fluorescein dye analogue for the molecular imaging of retinal inflammasomes. (B) InflammaProbe-2 binds to the cavity between the NACHT subdomains of NLRP3, stabilizing the closed conformation of NLRP3 inflammasome, allowing imaging of the NLRP3 inflammasomes. Molecular modeling was performed using PDB ID: 7PZC and AutoDock program. Based on the lowest-energy conformation, InflammaProbe-2 binds to the active site of the NACHT domain of NLRP3 inflammasome. See Supporting Information Figure S2 for more details.

## Results

### Design and Synthesis of InflammaProbe-**2**

The design of InflammaProbe-2 is optimized based on a well-characterized NLRP3 inhibitor, MCC950 (Figure 1). The design of this novel probe for molecular imaging includes clinically used components to minimize unwanted side effects on retinal cells and tissues. As illustrated in Figure 1, InflammaProbe-2 features a 2-furan sulfonylurea derivative conjugated via an amide linkage to a fluorescent dye compatible with fluorescein angiography equipment commonly used in the clinic. Since MCC950 has a modification at the B-position, as shown in Figure 1, we synthesized several compounds with modifications at the B-position. Modification at the A-position was excluded to avoid the possibility of steric hindrance of the imaging probe with the probe-binding sites. InflammaProbe-2 features modification at the C-position of the 2-furan ring, where the modification is furthest away from the binding site of the cavity between the NACHT subdomains of NLRP3. This C-position may contribute to stabilizing the closed conformation of NLRP3 inflammasome, allowing imaging of the NLRP3 inflammasomes.^31^

After synthesizing a library of compounds with modifications at the B- and C-positions of MCC950, as shown in Figure 2, compounds were tested for the inhibition of NLRP3-mediated secretion of IL-1β in retinal pigment epithelial cells (ARPE-19 cells). The data is shown in Figure 2. Levels of IL-1β were measured by performing ELISA as described in the Experimental Section. InflammaProbe-2, MCC950, and Compounds 1, 3, and 4 all significantly inhibited the LPS-primed and nigericin-stimulated production of IL-1β in ARPE-19 cells. Modification at both the B- and C-positions retained the bioactivity of the parent compound, MCC950. However, Compound 2, the precursor for InflammaProbe-2, showed minimal inhibitory activity, which may be due to a nonspecific interaction caused by the free amine group. InflammaProbe-2 is a potent IL-1β inhibitor. These data suggest that InflammaProbe-2 retains the inhibitory activity of its parent compound, MCC950, which enables it to target the NLRP3 inflammasomes.

**Figure 2:**
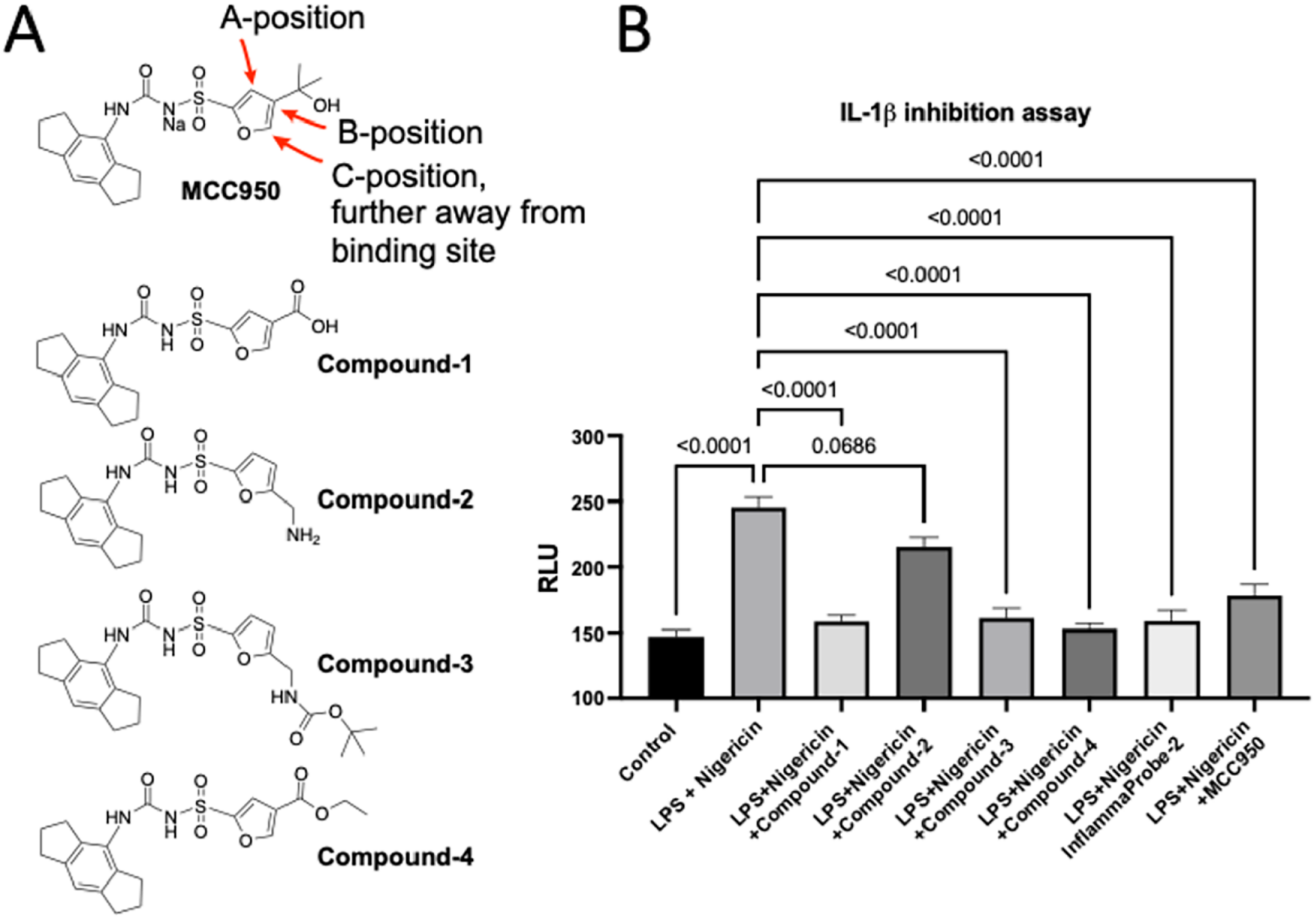
InflammaProbe-2 inhibits NLRP3-mediated secretion of IL-1β in ARPE-19 retinal pigment epithelial cells. (A) Chemical structures of MCC950 and its derivatives with modifications at the A-, B-, and C-positions. (B) InflammaProbe-2, MCC950, Compounds 1, 3, and 4 significantly inhibited LPS-primed and nigericin-stimulated production of IL-1β. Compound 2, the precursor for InflammaProbe-2, showed minimal inhibitory activity; however, InflammaProbe-2 showed potent IL-1β inhibition, suggesting that InflammaProbe-2 retains the inhibitory activity of the parent MCC950 and could target NLRP3 inflammasomes. ELISA was used to measure the levels of IL-1β in the treatment media. The data were expressed as the mean ± SD (*n* = 6). Statistical analysis was performed by one-way ANOVA; *P*-value <0.05 was considered statistically significant.

Next, we performed fluorescence properties of InflammaProbe-2 as shown in Figure 3. InflammaProbe-2 showed biocompatibility with ocular imaging and suitability for in vivo studies due to its high solubility in aqueous medium and high fluorescence properties. InflammaProbe-2 is a highly fluorescent small molecule, MW 770.8 g/mol, with an excitation maximum at 490 nm and emission maximum at 520 nm.

**Figure 3:**
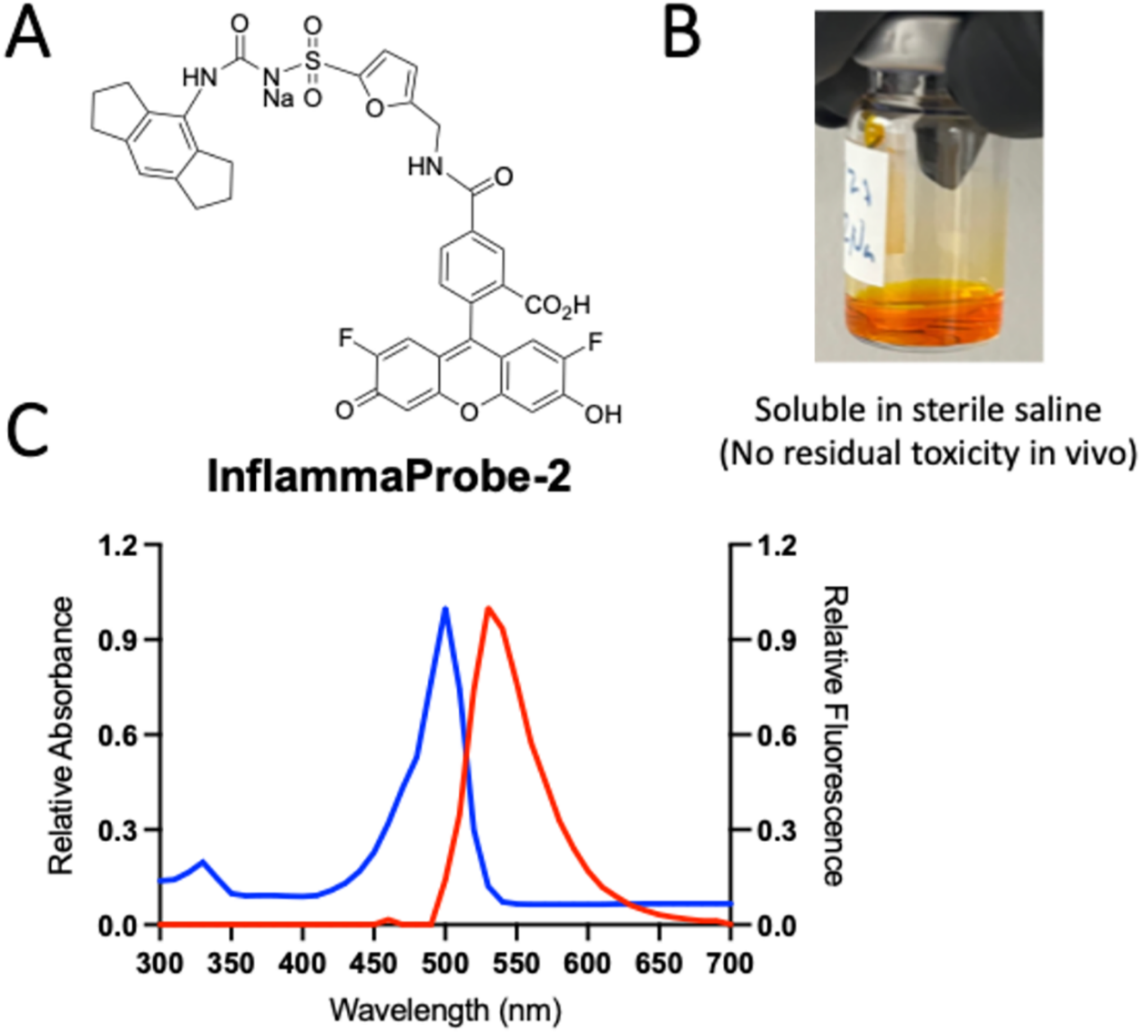
Chemical properties of InflammaProbe-2. (A) Chemical structure of InflammaProbe-2, a derivative of MCC950, suitable for the molecular imaging of NLRP3 inflammasomes using angiographic equipment. (B) InflammaProbe-2 is biocompatible with ocular tissues and suitable for in vivo studies due to its high solubility in aqueous medium and minimal toxicity to retinal cells. (C) InflammaProbe-2 is a highly fluorescent small molecule, MW 770.8 g/mol, with an excitation maximum at 490 nm and emission maximum at 520 nm.

### Role of NLRP3 Activation in Diabetic Retinopathy Progression

In diabetes, hyperglycemia may activate the NLRP3 inflammasome, causing apoptosis of retinal cells (Figure 4). We observed neurodegeneration and retinal ganglion cell (RGC) apoptosis in STZ-induced diabetic retina (Figure 4B). At an early onset of DR, sustained hyperglycemia activates NLRP3 inflammasomes, which may lead to pyroptosis or programmed cell death of pericytes and vascular endothelial cells in the retina, causing diabetic macular edema (DME) from vascular leakage or causing nonproliferative diabetic retinopathy (NPDR) from microvascular damage. Furthermore, activation of NLRP3 inflammasomes induces the production of proinflammatory cytokines, including IL-1β, that may lead to vascular endothelial growth factor (VEGF) production by the retinal cells, which may cause proliferative diabetic retinopathy or PDR. Thus, imaging NLRP3 inflammasomes in diabetic retina could provide important molecular information characterizing the risk of DME, NPDR, or PDR before the onset of these conditions.

**Figure 4:**
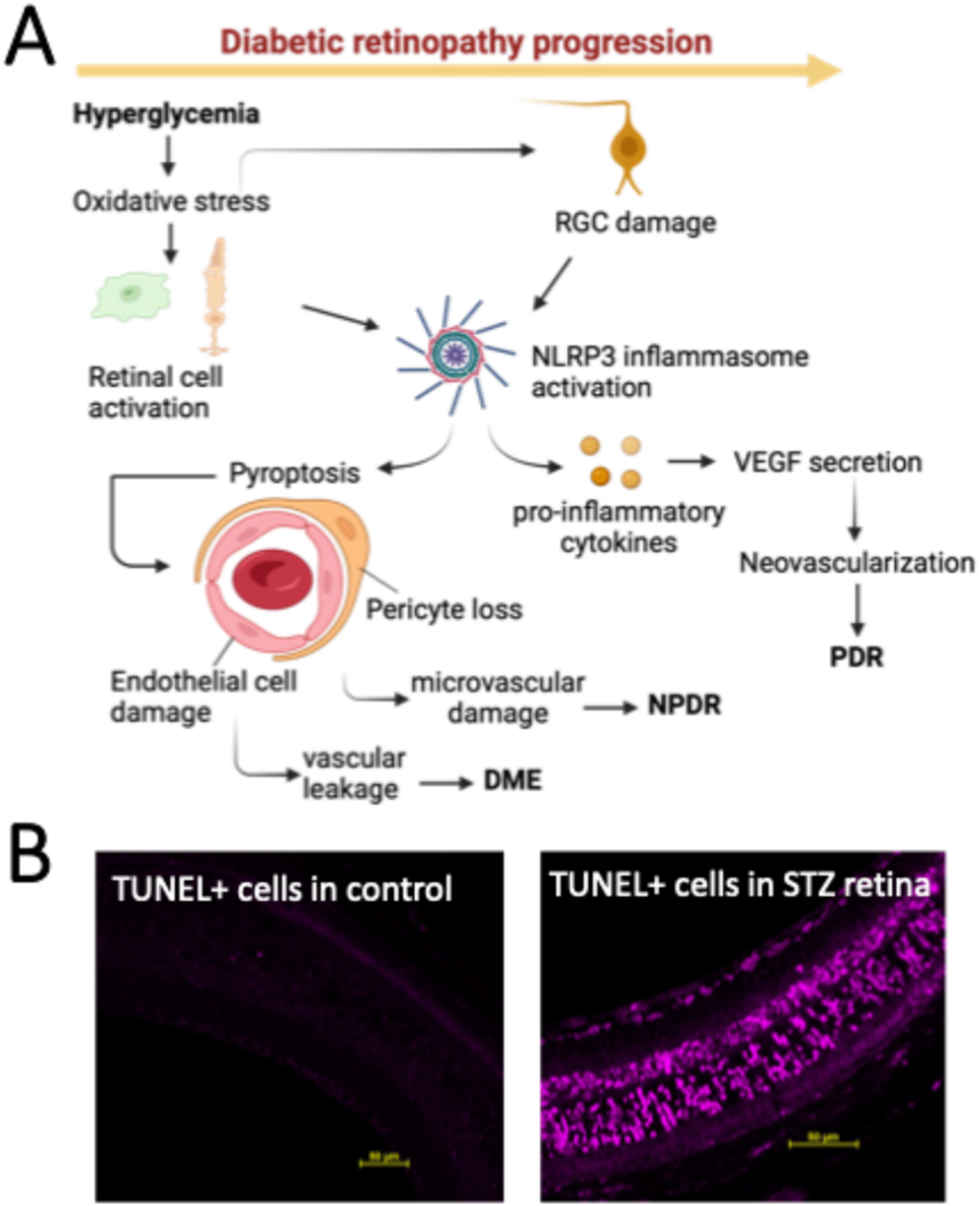
NLRP3 inflammasome activation may cause retinal cell apoptosis in diabetic retinopathy. (A) Schematic drawing showing hyperglycemia-induced programmed cell death in progressive diabetic retinopathy. Hyperglycemia may activate NLRP3 inflammasome, which may lead to programmed cell death, causing diabetic macular edema (DME) from vascular leakage, nonproliferative diabetic retinopathy (NPDR), and proliferative diabetic retinopathy (PDR). A drawing was created using BioRender.com. (B) TUNEL-positive apoptotic cells were detected in STZ retinas at 3 months postdiabetes induction. TUNEL-positive apoptotic cells were not detectable in age-matched healthy control retinas. Experiments were performed with three technical and three biological replicates. Scale bar 50 μm.

### Molecular Imaging of NLRP3 Inflammasomes in Human Retinal Cells In Vitro

Since NLRP3 localizes to the endoplasmic reticulum structures in the cytosol before activation and redistributes to the perinuclear space after activation,^32^ we assessed InflammaProbe-2′s ability to detect NLRP3 inflammasome activation in LPS-primed and nigericin-stimulated ARPE-19 cells. InflammaProbe-2 is highly efficient in the targeted visualization of NLRP3 inflammasome activations in human retinal cells treated under inflammatory conditions (Figure 5). Increased levels of InflammaProbe-2-dependent fluorescence were observed in LPS-primed and nigericin-stimulated cells (Figure 5E–G), presumably detecting activated NLRP3 inflammasomes. Interestingly, InflammaProbe-2 fluorescence was localized mostly at the perinuclear sites in activated ARPE-19 cells, as shown in Figure S3. Minimal levels of InflammaProbe-2 fluorescence were observed in the control cells (inactive NLRP3) and LPS-treated cells, where NLRP3 is primed but inactive (Figure 5A–D). Mechanistically, InflammaProbe-2 may bind to the cavity between the NACHT subdomains of NLRP3, stabilizing the closed conformation of NLRP3 inflammasome, allowing imaging of the NLRP3 inflammasomes.^31^ The specificity of InflammaProbe-2 was confirmed by blocking the receptor using the potent inhibitor, MCC950. InflammaProbe-2-derived fluorescence signals were decreased significantly in LPS-primed and nigericin-treated cells after blocking its target using MCC950 (Figure 5H–J). These results indicate that InflammaProbe-2 could selectively detect NLRP3 inflammasome activations and *not* the inactive NLRP3 in retinal cells.

**Figure 5:**
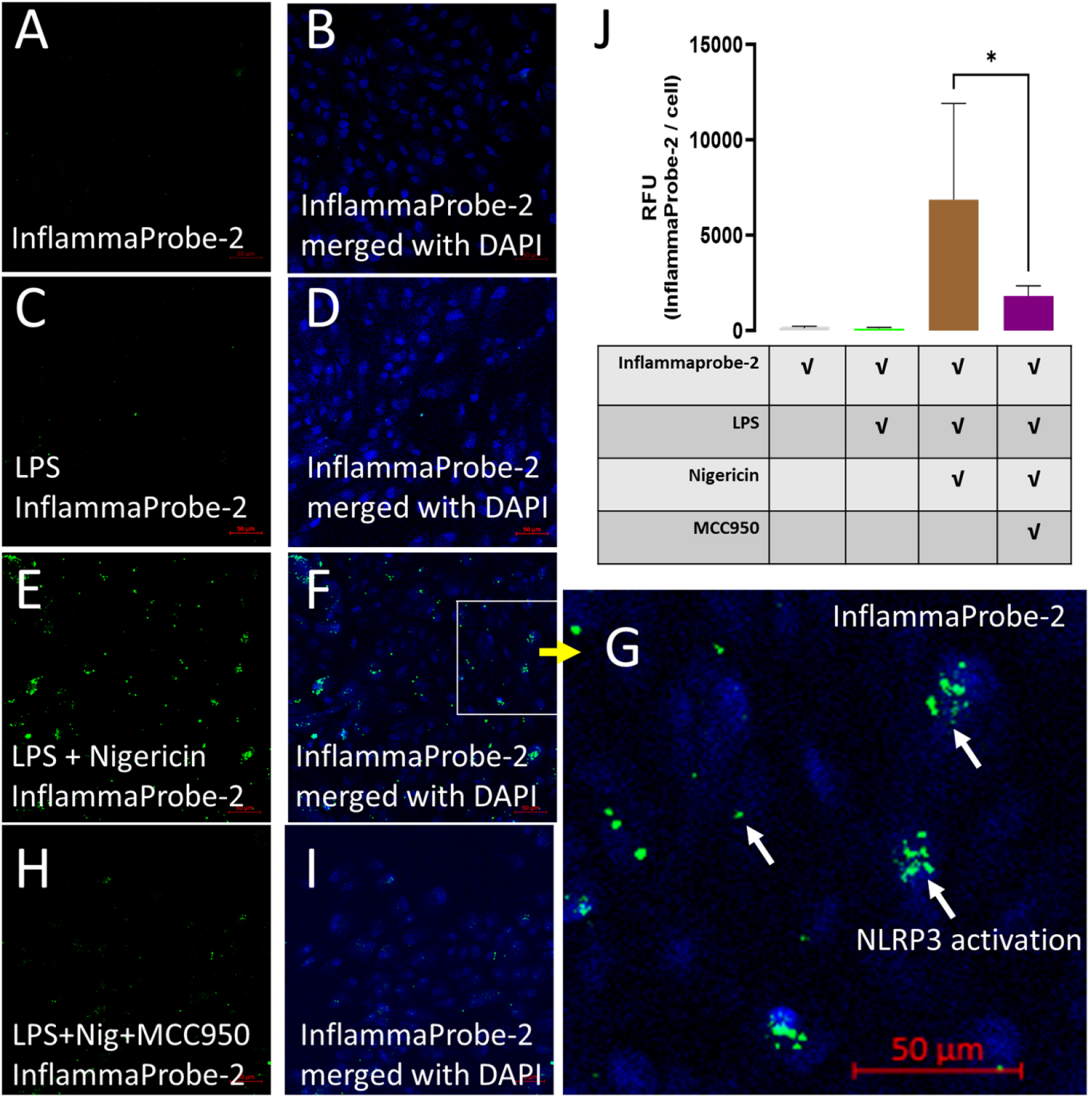
Confocal imaging of NLRP3 inflammasome activations in ARPE-19 cells using InflammaProbe-2. ARPE-19 cells were treated with InflammaProbe-2 under normal conditions (A, B), LPS-primed (C, D), and LPS-primed and nigericin-stimulated conditions (E, F), and imaged using confocal fluorescence microscopy. (G) Magnification area in (F). (H, I) To confirm specificity, InflammaProbe-2 fluorescence was monitored after blocking the NLRP3 inflammasome using MCC950. (J) Quantitative analysis of InflammaProbe-2 fluorescence in RFUs per cell using the confocal microscopy images. Relative to the untreated control and LPS-primed cells, increased levels of InflammaProbe-2-dependent fluorescence were observed in LPS-primed and nigericin-treated cells. These fluorescence signals were decreased significantly after blocking its target using MCC950. These results indicate that InflammaProbe-2 could selectively detect NLRP3 inflammasome activations in retinal cells. Scale bar in images (A–G) was 50 μm, and statistical significance was *P < 0.05.

### Hyperglycemic Activation of NLRP3 Inflammasomes and Imaging in Human Retinal Cells

In diabetic retinopathy, hyperglycemia may initiate NLRP3 inflammasome activation.^33^ To understand the role of hyperglycemia in NLRP3 inflammasome activation in diabetes, we exposed human retinal pigment epithelial cells (ARPE-19) to hyperglycemic conditions by treating them with 30 and 50 mM d-glucose and compared them with normoglycemic control ARPE-19 cells. We observed that hyperglycemia did not change the expression of NLRP3 mRNA at either 30 mM and 50 mM d-glucose-treated ARPE-19 cells compared to normoglycemic control (Figure 6A). However, NLRP3 inflammasome activation was observed in both 30 and 50 mM d-glucose-treated ARPE-19 cells as monitored by IL-1β expression in hyperglycemic cells compared with normoglycemic control cells (Figure 6B). NLRP3 activation may also contribute to the programmed cell death in diabetic retinopathy (Figure 4B). To monitor the sensitivity of InflammaProbe-2 in detecting NLRP3 inflammasome activation in hyperglycemic conditions, we have monitored InflammaProbe-2-derived fluorescence in ARPE-19 cells treated under hyperglycemic conditions (Figure 6C–G). Strong fluorescence was observed in hyperglycemic ARPE-19 cells, which were statistically significant (*P* < 0.0001) compared to cells treated under normoglycemic conditions (Figure 6G).

**Figure 6:**
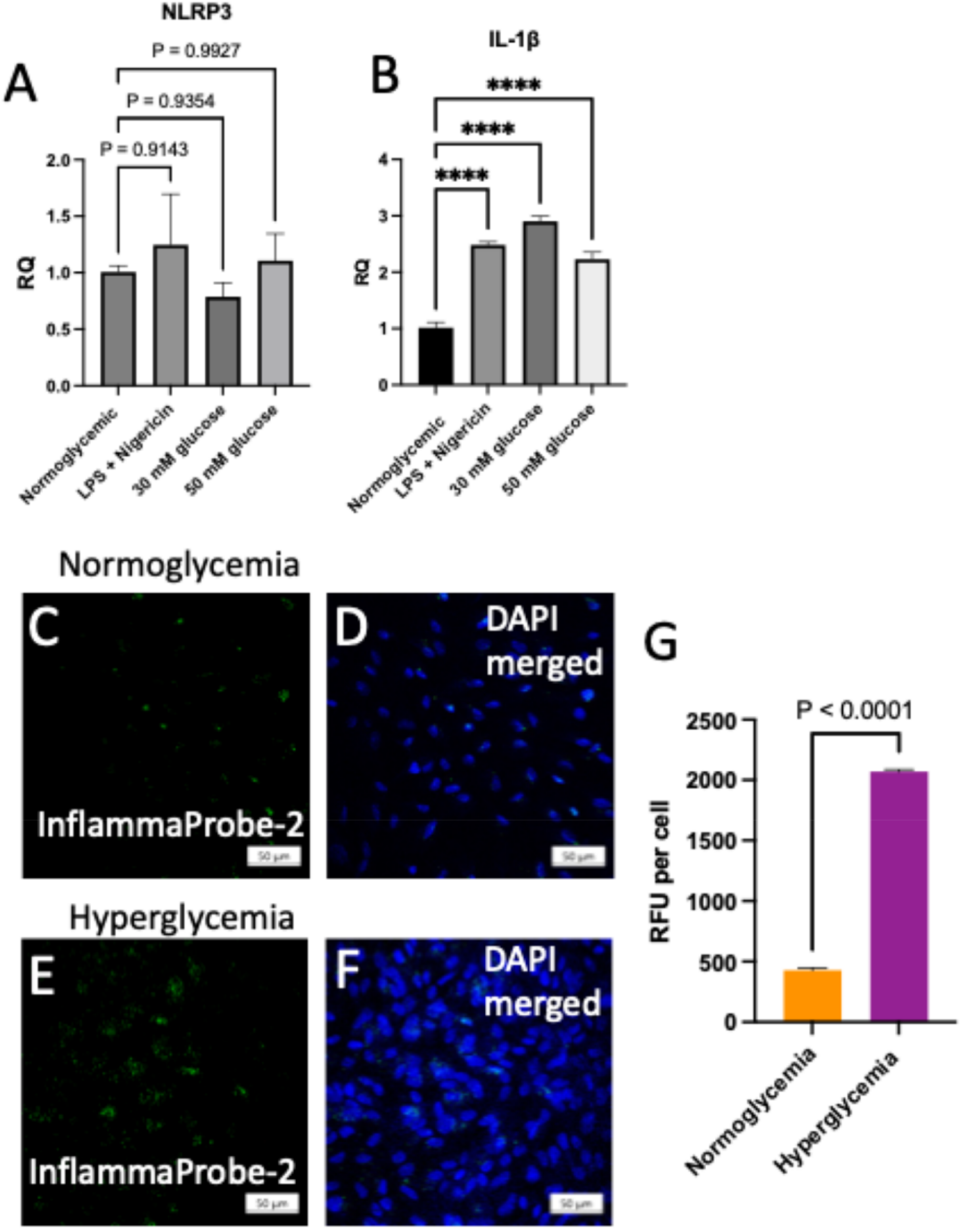
Imaging NLRP3 inflammasome activations in hyperglycemic ARPE-19 cells. (A, B) NLRP3 activation was confirmed by monitoring the expression of IL-1β in hyperglycemic ARPE-19 cells. ARPE-19 cells were treated under normoglycemic conditions and hyperglycemic conditions (30 and 50 mM d-glucose). In addition, the inflammatory condition (LPS + nigericin) was used as a positive control to compare the expression of IL-1β in ARPE-19 cells. (C, D) ARPE-19 cells were treated with InflammaProbe-2 under normoglycemic conditions and hyperglycemic conditions (E, F) and imaged using confocal fluorescence microscopy. (G) Quantitative analysis of InflammaProbe-2 fluorescence in RFUs per cell using the confocal microscopy images. Scale bar in images (C–F) was 50 μm, and statistical significance was ****P < 0.0001.

To further explore the sensitivity of InflammaProbe-2, time- and dose-dependent changes in fluorescence were monitored over time in ARPE-19 cells treated under different glucose treatment conditions. We observed that InflammaProbe-2 fluorescence increased with increases in d-glucose concentration (Figure 7). These changes in fluorescence may represent levels of NLRP3 inflammasome activation in activated cells.

**Figure 7:**
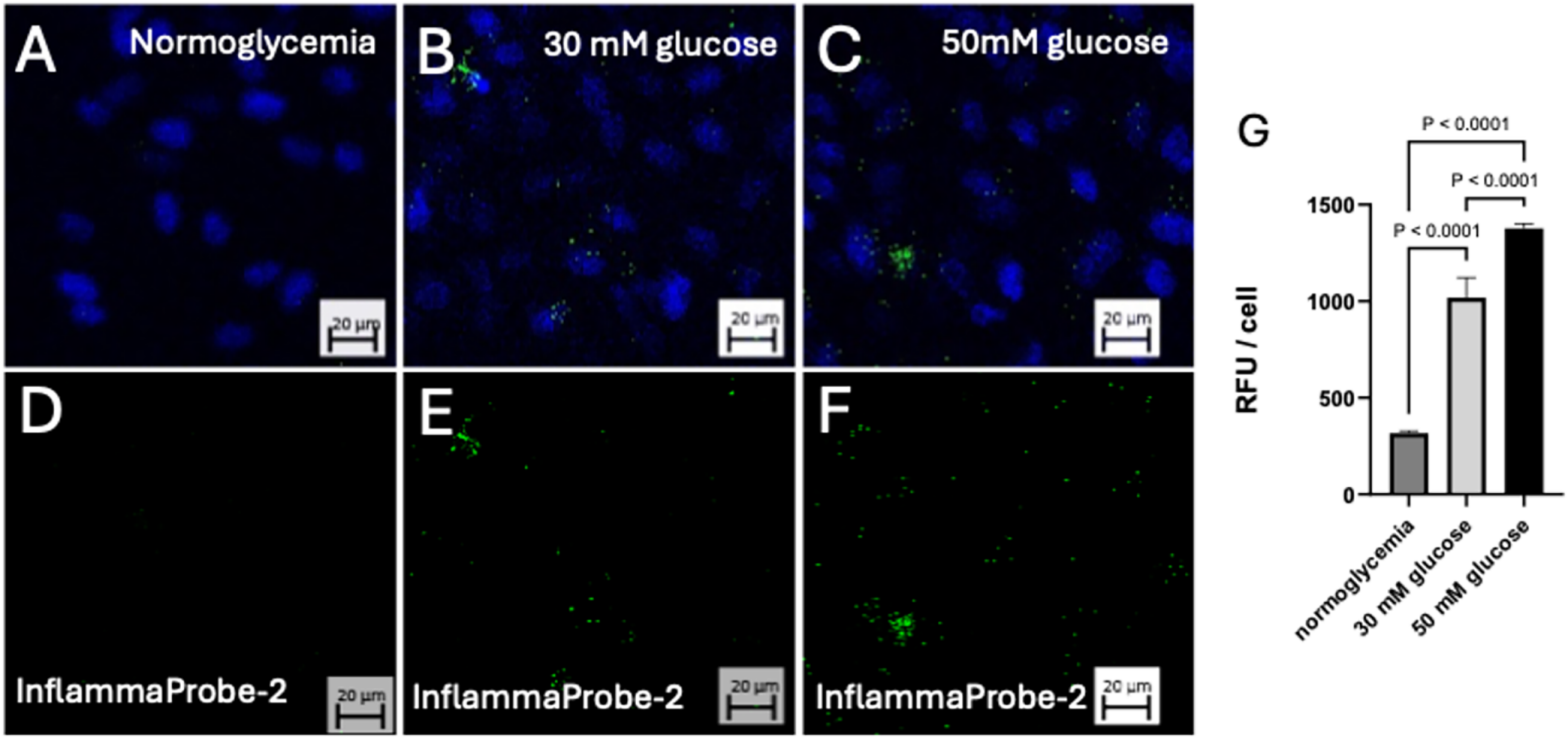
Intensity of the InflammaProbe-2 signal increases with gradient d-glucose concentrations. ARPE-19 cells were incubated in media of varying d-glucose concentrations for 3 h in the presence of 10 μM of InflammaProbe-2. (A, D) Normoglycemic cells showed minimal InflammaProbe-2 fluorescence. (B, E) Cells incubated in 30 μM media showed increased InflammaProbe-2 fluorescence compared to cells under normoglycemic conditions. (C, F) Cells incubated in 50 μM d-glucose had the highest InflammaProbe-2 signal of all three glucose concentrations. These are representative images from the minimum *n* = 3 replicates.

Although the InflammaProbe-2 fluorescence signal increased over time for 3 h, the fluorescence signal decreased significantly at the 6 h time point compared to that at 3 h (Figure S4). Our results indicate that the activation of NLRP3 inflammasome and high-glucose-induced programmed cell death are closely related. We hypothesized that the activation of NLRP3 inflammasome requires time at the early stage for its activation. After activation, the activated NLRP3 inflammasome may initiate the programmed cell death, which is reflected in the 6 h treatment group. We are currently investigating the role of hyperglycemia in NLRP3 inflammasome activation and programmed cell death in the diabetic retina.

Furthermore, InflammaProbe-2 showed distinct sensitivity based on the treatment. We compared the sensitivity of InfammaProbe-2 in LPS-primed and nigericin-activated ARPE-19 cells with that of cells induced with hyperglycemic conditions. We observed that InflammaProbe-2 fluorescence was significantly higher in LPS-primed and nigericin-treated ARPE-19 cells compared to activated cells induced with large amounts of glucose (Figure 8). These data indicate that levels of NLRP3 inflammasome activation is higher in LPS-primed nigericin treated cells compared to high glucose treated cells. Thus, InflammaProbe-2 could reliably detect levels of NLRP3 inflammasome activation in different treatment groups.

**Figure 8:**
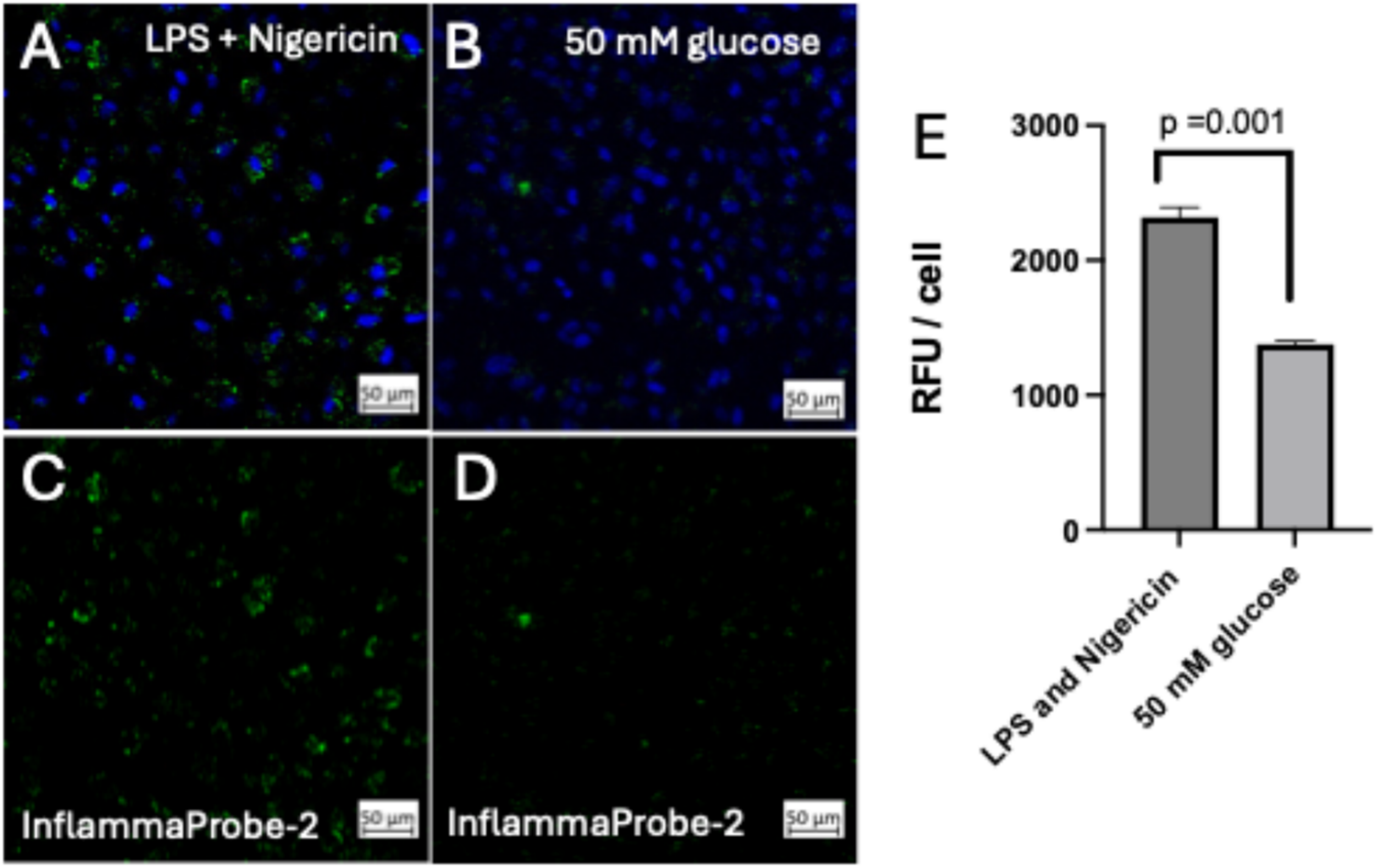
InflammaProbe-2 shows distinct sensitivity based on the treatment. (A, C) ARPE-19 cells were treated with 500 ng/mL of LPS overnight and then concurrently for 3 h with nigericin (20 μM) and InfammaProbe-2 (10 μM). Confocal images were captured, and fluorescence intensities were calculated using ImageJ software. (B, D) ARPE-19 cells were treated with media containing 50 mM d-glucose. At 3 h post-treatment, InflammaProbe-2 was added to the media (10 μM) and incubated for an additional 3 h. Confocal images were captured, and fluorescence intensities were calculated using ImageJ software. (E) The InflammaProbe-2 fluorescence signals were significantly higher in LPS-primed and nigericin-treated ARPE-19 cells compared to high-glucose-induced activated cells (*P* = 0.001). These data indicate that InflammaProbe-2 could reliably detect levels of NLRP3 inflammasome activation in different treatment groups. These are representative images from a minimum of *n* = 3 replicates.

### In Vivo Molecular Imaging of NLRP3 Inflammasomes in Mouse STZ Retinas

In vivo molecular imaging of NLRP3 inflammasome activation was performed in mouse STZ. InflammaProbe-2 was injected intraperitoneally (10 mg/kg) in mouse STZ and age-matched controls. In vivo fluorescence imaging was performed four hours post-InflammaProbe-2 injection. InflammaProbe-2-dependent strong fluorescence was observed in STZ-retina compared to the nondiabetic controls (Figure 9). As expected, InflammaProbe-2-dependent fluorescence was observed as distributed across the retina, similar to the distribution of apoptotic cells in the STZ retinas, as shown in Figure 4B. The specificity of InflammaProbe-2 was confirmed in vivo by blocking its receptor using the parent compound inhibitor, MCC950. InflammaProbe-2-derived fluorescence signals were significantly decreased in STZ retinas after blocking its target using MCC950 (Figure 9E–G). These results indicate that InflammaProbe-2 could selectively detect NLRP3 inflammasome activations in vivo in diabetic retina. For blocking experiments, a separate group of mice received intraperitoneal injections of MCC950 hydrochloride at a concentration of 20 mg/kg body weight and were treated for 2 h; they were then injected with InflammaProbe-2 at a concentration of 10 mg/kg. In vivo images were captured at four hours post-InflammaProbe-2 injection.

**Figure 9:**
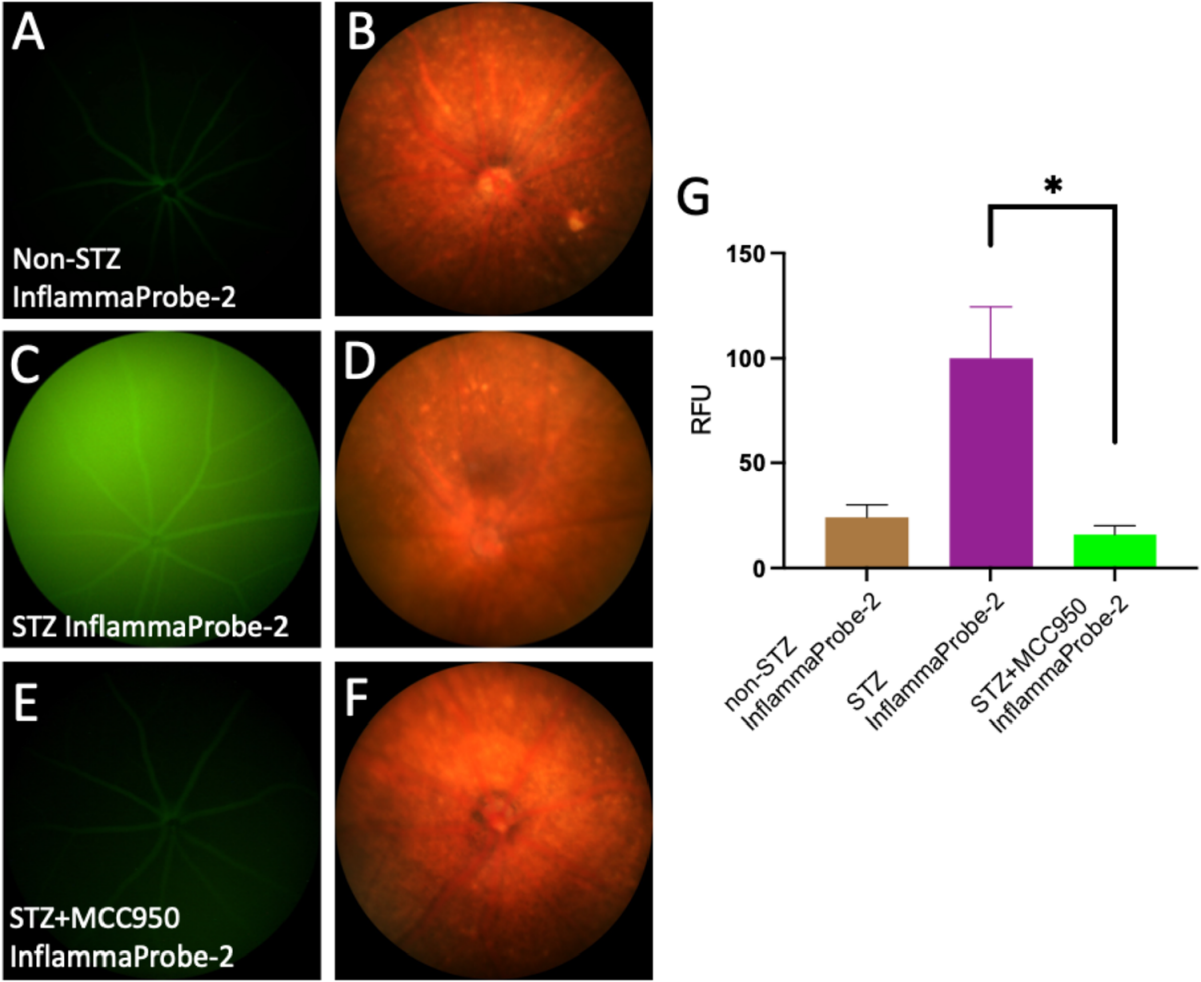
In vivo molecular imaging of NLRP3 inflammasome activation in STZ-induced diabetic retina using InflammaProbe-2. Fluorescence and brightfield fundus images were captured 4 h after 20 mg/kg intraperitoneal injection of InflammaProbe-2 in nondiabetic animals (A, B), STZ-induced diabetic animals (C, D), and STZ animals pretreated with MCC950 (E, F). (G) Quantification of fluorescence intensities showed that relative to the nondiabetic control retinas, increased levels of InflammaProbe-2-dependent fluorescence were observed in diabetic retinas, which were blocked by MCC950. These results indicate that InflammaProbe-2 selectively detects NLRP3 inflammasome activations. Data are representative of six replicates from each experimental group. Statistical significance *P < 0.05.

### Ex Vivo Colocalization of InflammaProbe-2 and NLRP3 in Mouse STZ Retinas

NLRP3 and InflammaProbe-2 fluorescence were characterized in mouse STZ retinas and compared with nondiabetic controls. After in vivo imaging, confocal imaging was performed in the same STZ retinas and nondiabetic controls as those used for in vivo imaging. Distribution of NLRP3 in the STZ retinas was confirmed by strong fluorescence from NLRP3 immunostaining in transverse STZ-retinal cross sections compared to nondiabetic control retinas (Figure 10A,B). In addition, InflammaProbe-2-dependent strong fluorescence was observed laterally across the entire retina, indicated by the fluorescence signals, compared to the nondiabetic controls, suggesting the targeted visualization of NLRP3 inflammasomes in the STZ retinas (Figure 10C,D). The InflammaProbe-2 fluorescence patterns were similar to the patterns observed in NLRP3 immunostaining, suggesting the targeted visualization of NLRP3 inflammasomes by the imaging probe.

**Figure 10:**
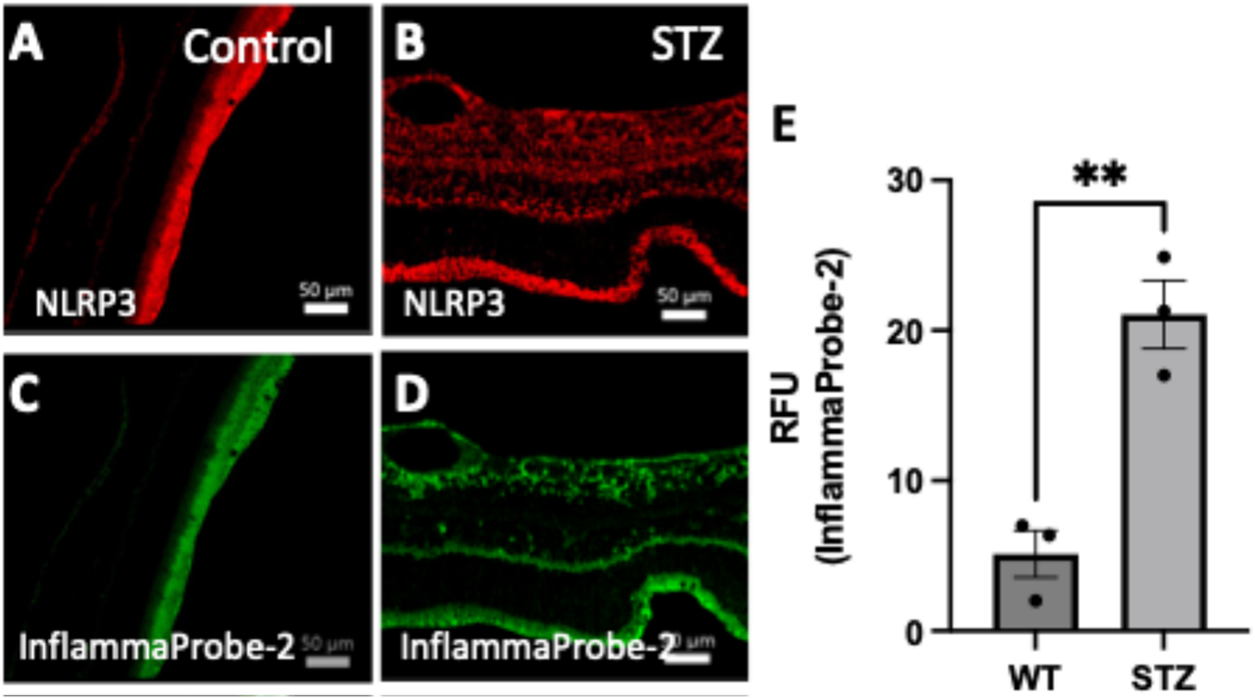
Confocal imaging of NLRP3 inflammasomes in STZ retinas and nondiabetic WT controls. The STZ-induced diabetic mice and age-matched WT control mice received intraperitoneal injections of InflammaProbe-2 (20 mg/kg). Tissues were analyzed for InflammaProbe-2 localization in retinal cross sections and costained with fluorescently tagged antibodies against NLRP3. The stained retinas were then imaged using confocal fluorescence microscopy at 20× magnification. (A, B) Increased levels of NLRP3 were observed in STZ-retinal cross sections compared to nondiabetic WT controls. (C, D) In addition, increased levels of InflammaProbe-2 fluorescence were localized in the diabetic retinal cross sections compared to nondiabetic controls. (E) Quantitative fluorescence intensities showed increased InflammaProbe-2 fluorescence in diabetic retinas compared to nondiabetic controls. These results indicate that InflammaProbe-2 selectively detects NLRP3 inflammasomes. Data are representative of three replicates from each experimental group. Statistical significance **P < 0.001.

### Toxicity of InflammaProbe-**2**

The toxicity of InflammaProbe-2 was assessed in vitro and in vivo. The cytotoxicity of InflammaProbe-2 was assessed in human ARPE-19 cells by using a cell viability assay. Cell viability was monitored after 24 h of treatment with 0.1–100 μM InflammaProbe-2. Cell viability was not significantly reduced in the highest InflammaProbe-2 treatment, 100 μM, compared to MCC950 and untreated controls (Figure 11A). We observed the cytotoxicity of MCC950 at 100 μM concentration, consistent with the reported toxicity of MCC950 at this concentration.^34^ In vivo toxicity was also assessed in dark-adapted animals using Ganzfeld electroretinography (ERG) 2 weeks after the intraperitoneal injection of InflammaProbe-2 at 20 mg/kg. No significant changes in a-wave and b-wave amplitudes of their electrical response to a 100 cd·s/m^2^ light flash were observed as compared to vehicle-treated mice (Figure 11B). These results suggest that InflammaProbe-2 is minimally toxic to primary cells or retinal tissues. To further confirm any residual toxicity of InflammaProbe-2 to retinal tissues, a TUNEL assay was performed in retinal cross sections. Tissues were analyzed 2 weeks after intraperitoneal injection of InflammaProbe-2 (20 mg/kg). InflammaProbe-2-treated retinal cross sections showed no cellular damage in TUNEL-stained tissues (Figure 11C–H). Furthermore, InflammaProbe-2 concentrations in whole blood were measured over time (Figure S5). Mice were injected with InflammaProbe-2 (10 mg/kg). Blood samples were analyzed to quantify the amount of InflammaProbe-2 in the blood circulation at different time points. The data indicate that InflammaProbe-2 is almost cleared from blood circulation after 3 h.

**Figure 11:**
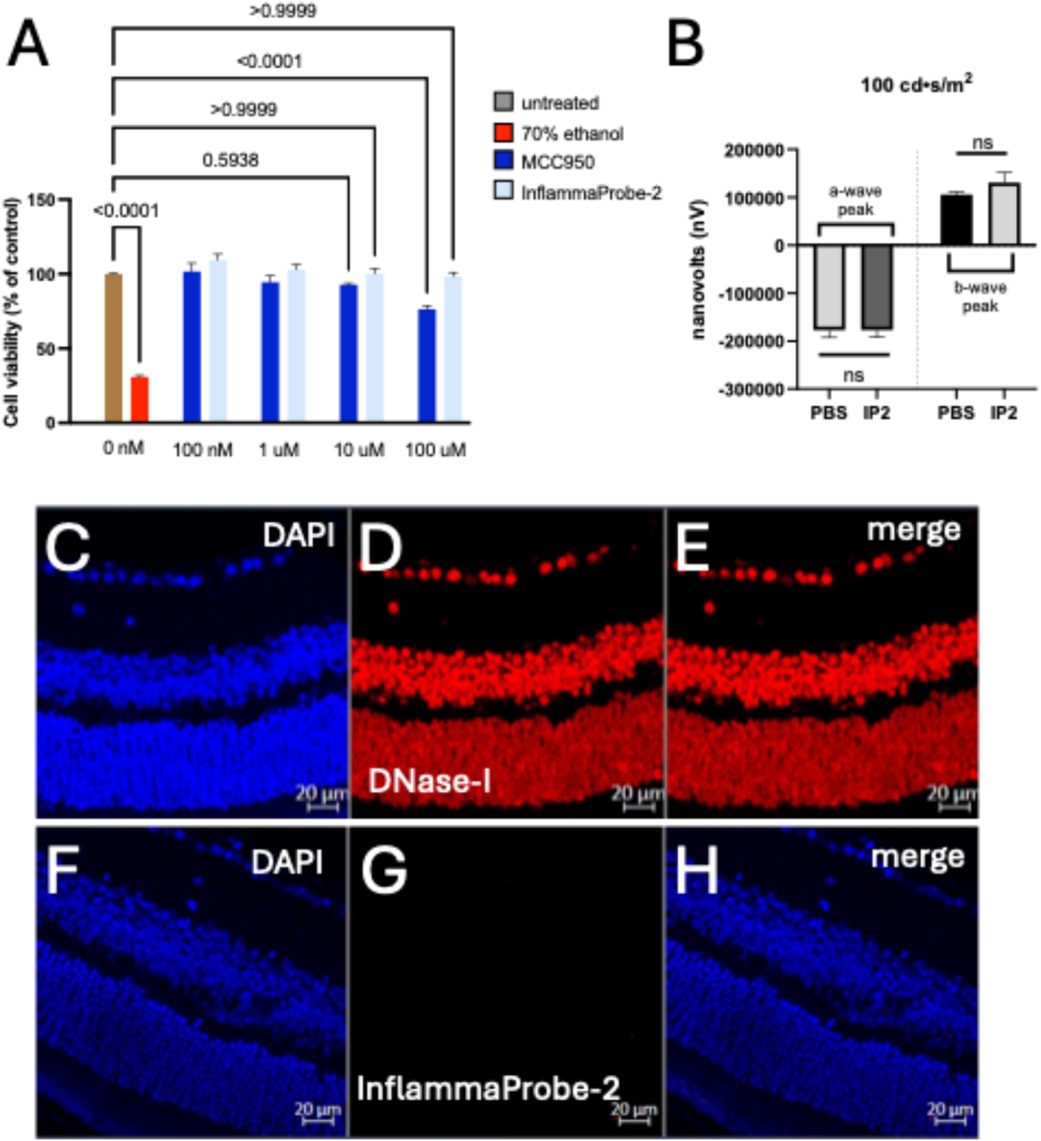
Toxicity of InflammaProbe-2 (IP2). (A) Cytotoxicity of InflammaProbe-2 was assessed in human ARPE-19 cells using a fluorescence-based assay using Calcein Deep Red AM ester. The viability of ARPE-19 cells was monitored after treatment with 0.1–100 μM InflammaProbe-2. After 24 h of treatments, cell viability was not significantly reduced in the highest InflammaProbe-2 concentration used, 100 μM. We observed the cytotoxicity of MCC950 at a 100 μM concentration, consistent with the literature.^34^ (B) In vivo toxicity was also monitored in dark-adapted animals using Ganzfeld electroretinography (ERG) 2 weeks after the intraperitoneal injection of InflammaProbe-2 at 20 mg/kg. InflammaProbe-2 showed no significant changes in a-wave and b-wave amplitudes of their electrical response to a 100 cd·s/m^2^ light flash, suggesting that InflammaProbe-2 is minimally toxic to the retina. Cell viability was expressed as the mean ± SD of 10 replicates from each group. Statistical analysis was performed by using one-way ANOVA; *P*-value of <0.05 was considered statistically significant. ERG data were expressed as mean ± SD of 6 retinas from each group (ns = not significant). (C–H) Toxicity of InflammaProbe-2 on retinal tissues was assessed using the TUNEL assay. The TUNEL assay was performed in retinal cross sections to assess retinal cell damage, and was taken as a measure of retinal toxicity. Tissues were analyzed 2 weeks after intraperitoneal injection of InflammaProbe-2 (20 mg/kg). (C) DAPI staining of nuclei. (D) DNase 1-treated retinal transverse sections served as a positive control. Fragmented DNA was clearly visible. (E) Merged image of (C) and (D). (F) DAPI staining of nuclei. (G) InflammaProbe-2-treated retinal cross sections showed no cellular damage. (H) Merged image of (F) and (G). TUNEL assay was performed in 3 retinas from each group.

Also, the stability of InflammaProbe-2 in blood serum was monitored over time, and the data are shown in Figure S6. InflammaProbe-2 was incubated with blood serum at 37 °C and analyzed at different time points (10 min, 1, 8, 12, 24 h). We observed that InflammaProbe-2 was stable in serum medium even after 8 h. Almost the entire InflammaProbe-2 remained as an intact probe after 8 h incubation in serum medium at 37 °C. The LCMS data showed a metabolite with an LRMS (ESI) *m*/*z* of 530.87 after 12 h incubation in serum medium at 37 °C (Figure S6).

## Discussion and Conclusions

Diabetic retinopathy is a leading cause of vision loss affecting a large number of the working-age population worldwide. The NLRP3 inflammasome activation may initiate pyroptotic cell death in diabetic retinopathy.^8^ We hypothesized that in vivo molecular imaging of NLRP3 inflammasomes would create a platform to detect the early onset of diabetic retinopathy before any permanent damage to the retina, such as pyroptotic cell death in diabetic retinopathy. We have synthesized a novel molecular imaging probe, InflammaProbe-2, for the in vivo molecular imaging of NLRP3 inflammasome activations in the living retina. InflammaProbe-2 was able to detect NLRP3 inflammasomes in vitro and in vivo. InflammaProbe-2 is highly sensitive to detect NLRP3 inflammasomes and is safe for retinal cells and tissues.

Our first-generation InflammaProbe-1 was designed based on CY09, and it was bound to both activated and inactivated NLRP3. The next-generation InflammaProbe-2 is designed based on MCC950, which binds to the NACHT domain and could detect only activated inflammasomes in cultures of retinal cells and also in the living retina. We have used a commercially available Micron IV in vivo imaging system, and the resolution was limited to the resolved individual NLRP3 inflammasomes as fluorescence puncta. However, our ex vivo data clearly demonstrate that the NLRP3 inflammasomes are activated in multiple cell types in the diabetic retina, as shown in Figure 4. Patterns of apoptotic cells in STZ retinas shown in Figure 4, InflammaProbe-2 fluorescence, and NLRP3 staining in Figure 10 are very similar, suggesting that InflammaProbe-2 is capable of the targeted visualization of NLRP3 inflammasomes and pyroptosis in the living diabetic retina. Although our low-resolution Micron IV imaging system could detect InflammaProbe-2 fluorescence, single inflammasome imaging is very much limited using this imaging system. An improved AO-SLO or other high-resolution imaging system could overcome this limitation for clinical applications.

### Plausible Mechanism of the Action of InflammaProbe-**2**

Mechanistically, NLRP3 inflammasomes regulate caspase-1-mediated programmed cell death (pyroptosis), and imaging NLRP3 inflammasomes could provide an indirect method for imaging programmed cell death in the living retina (Figure 12). Our newly synthesized imaging probe, InflammaProbe-2, binds to NLRP3 inflammasomes in vivo, allowing molecular imaging of NLRP3 inflammasomes in the living retina. InflammaProbe-2 fluorescence in the retinal cross section, as shown in Figure 10, suggests that NLRP3 inflammasomes are localized in cells in the inner retinal layers of the STZ animals.

**Figure 12:**
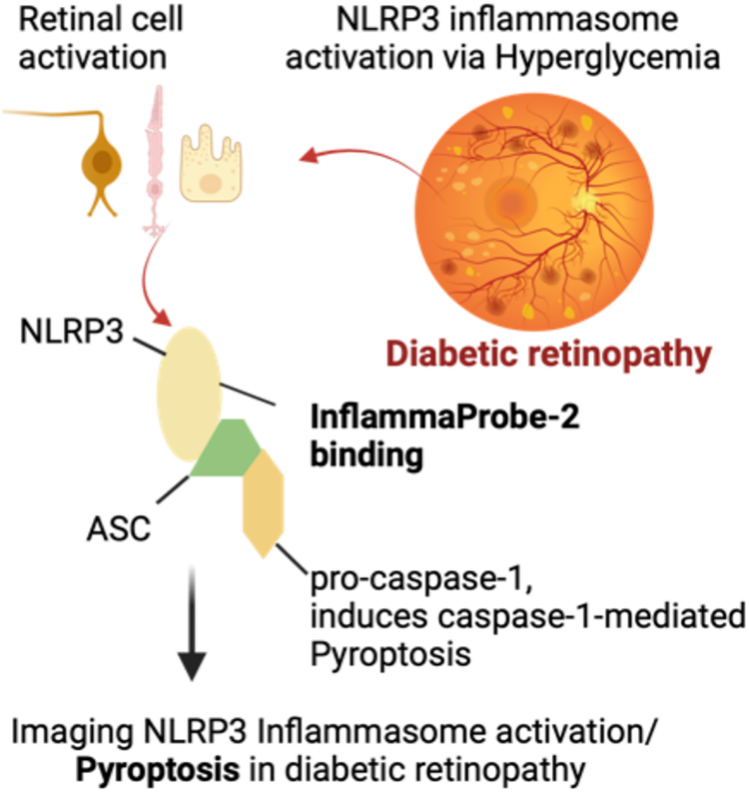
Plausible mechanism of action of InflammaProbe-2. Chronic low-grade inflammation plays an essential role in the development and progression of diabetic retinopathy (DR). Activation of NLRP3 inflammasomes has been associated with DR progression. Thus, imaging NLRP3 inflammasomes may provide a platform to detect the early onset of DR and monitor treatment response. To this end, we have synthesized an imaging probe, InflammaProbe-2, that binds to NLRP3 inflammasomes in vivo, allowing molecular imaging of NLRP3 inflammasomes in the living retina. In addition, mechanistically, NLRP3 inflammasomes regulate the caspase-1-mediated programmed cell death (pyroptosis), so imaging NLRP3 inflammasomes could provide an indirect method to image programmed cell death in the living retina.

In summary, we have developed a novel molecular imaging probe, InflammaProbe-2, for the direct imaging of NLRP3 inflammasome activations in the living retina. We have confirmed the specificity and sensitivity of InflammaProbe-2 for the detection of NLRP3 inflammasomes in a living diabetic retina using an animal model of diabetic retinopathy. These studies have significant potential for advancing the development of molecular imaging methods to detect early disease onset in the diabetic retina at a molecular level and monitor disease progression and response to therapy.

## Experimental Section

### Cells and Reagents

The human retinal pigment epithelial cell line, ARPE-19, was purchased from ATCC (Manassas, VA). The cells were maintained in a humidified environment with 5% CO_2_ at 37 °C unless otherwise noted. The ARPE-19 cells were cultured in DMEM/F12 (1:1) (Gibco, 11320–033) cell culture media (normoglycemic) with l-glutamine and 15 mM HEPES (Gibco, catalog #11330–32) with added Antibiotic-Antimycotic (100×, Gibco catalog # 15240–062). The cells were cultured in complete media supplemented with 2% fetal bovine serum (FBS) and maintained at 70–80% confluency in 0% FBS during all treatments unless otherwise noted.

### Animals

The C57BL/6 mice were purchased from Charles River Laboratories, Chicago, Illinois. All animal experiments were performed by following the protocols evaluated and approved by the Vanderbilt University Institutional Animal Care and Use Committee (Ethics Approval Number: M1800123) and were performed in accordance with the ARVO Statement for the Use of Animals in Ophthalmic and Vision Research and in compliance with ARRIVE guidelines. At the time of diabetes induction, the C57BL/6 mice were 6–8 weeks old. The mice were group-housed according to their randomly assigned experimental groups in ventilated cages maintained under a 12 h light and dark cycle at 22 ± 2 °C within an institutional animal care facility. The mice were provided with clean water (Nashville Metro Water Services, Nashville, TN) and a standard diet consisting of 4.5% fat (PicoLab Rodent Diet 5L0D; LabDiet, St. Louis, MO) ad libitum. The mice were sacrificed by CO_2_-induced asphyxiation followed by cervical dislocation.

### Anesthesia, Pupillary Dilation, and Corneal Numbing

Anesthesia, pupillary dilation, and corneal numbing were performed according to our previously published method.^35^ Anesthesia, pupillary dilation, and corneal numbing were performed prior to the following procedures: in vivo imaging and electroretinography (ERG). The mice were injected i.p. with a 70 μL mixture of ketamine (85.7 mg/kg) and xylazine (17.9 mg/kg) in sterile saline (1:1:2 mixture). The mice were an average of 22-g-weight mice (n = 12 per group). Pupils were dilated in anesthetized animals with a drop of 0.5% tropicamide and 2.5% phenylephrine. In addition, a drop of 0.5% proparacaine was used for corneal numbing. A GenTeal gel was used to maintain hydration during in vivo imaging. For ERG measurements, the eyes were hydrated by using artificial tears.

### Imaging NLRP3 Inflammasomes in LPS- and Nigericin-Treated ARPE-19 Cells

ARPE-19 cells were cultured in 4-well chamber slides (Lab-Tek, catalog no. 154526, Rochester, NY) and treated with 500 ng/mL LPS overnight. Nigericin was then added (20 μM), and the cells were incubated for 3 h with or without Inflammaprobe-2 (10 μM). For blocking experiments, MCC950 (200 mM) was added to a complete medium with nigericin. After an hour of blocking with MCC950, Inflammaprobe-2 (10 μM) was then added to the cells and incubated for an additional 2 h. Then, the cells were washed twice with PBS, fixed for 10 min with 10% neutral buffer formalin (NBF), washed once more with PBS, and mounted using mounting media with DAPI (Invitrogen, P36971). Confocal images were captured using a Zeiss LSM 710 AxioObserver microscope (Carl Zeiss AG, Oberkochen, Germany) using an EC Plan-Neofluar 20×/0.50 M27 objective. The InflammaProbe-2 fluorescence intensity of each image was expressed as RFU per cell, which was calculated by dividing the raw integrated density, measured using the Fiji ImageJ2 software, by the number of DAPI-stained nuclei present in the image. The data were representative of four replicates per group.

### Imaging NLRP3 Inflammasomes in Hyperglycemic and Normoglycemic ARPE-19 Cells

Hyperglycemic media was generated by adding d-glucose (Fisher Scientific, catalog #50–488–677) to the normoglycemic DMEM/F12 (1:1) (Gibco, 11320–033) media to make both 30 and 50 mM high d-glucose media. ARPE-19 cells were cultured following the method described above. The cells were either cultured under hyperglycemic or normoglycemic conditions with Inflammaprobe-2 and treated for 3 h. Then, the cells were washed twice with PBS, fixed for 10 min with 10% neutral buffer formalin (NBF), washed once more with PBS, and mounted using Prolong Diamond Antifade Mountant with DAPI (Invitrogen, P36971). Confocal images were captured using a Zeiss LSM 710 AxioObserver microscope (Carl Zeiss AG, Oberkochen, Germany) using EC Plan-Neofluar 20×/o.50 M27 objective. The InflammaProbe-2 fluorescence intensities were expressed as RFU per cell using the Imaris software. The data were representative of four replicates per group.

### ELISA

ARPE-19 cells were cultured in sterile 24-well plates (Fisher Scientific, FB012929) and treated with 500 ng/mL LPS overnight. Nigericin (20 μM) and InflammaProbe-2 or other related compounds (10 μM) were added to cells and incubated for 3 h. The media were collected after 3 h, and the IL-1β concentration was measured using the Promega Human IL-1β kit (catalog no. W6010), following the manufacturer’s protocol. Briefly, 50 μl of the media was transferred to a nonsterile white 96-well plate (Thermo Scientific, catalog no. 236108) and added in 1:1 to a freshly prepared 2× antibody solution from the kit (Promega Corporation, W117A/W118A). The media were incubated with antibodies for 60 min at room temperature. Twenty-five microliters of the detection substrate (Promega Corporation, VB405A) diluted in a detection buffer (Promega Corporation, VB406A) was added to each well and incubated at room temperature for 5 min. Immediately following incubation, luminescence was measured using a Cytation 5 plate reader (Agilent BioTek, Santa Clara, CA).

### Real-Time Quantitative PCR (qRT-PCR) Analysis

The qRT-PCR analysis was performed to measure NLRP3 and IL-1β mRNA in hyperglycemia and inflammatory conditions by following our previously published method.^25^ ARPE-19 cells were cultured in 6-well plates and cultured under growth conditions: normoglycemic, hyperglycemic (30 and 50 mM d-glucose), and inflammatory conditions (LPS + nigericin) as described above. After the treatment, the cells were washed with cold PBS (Life Technologies) and the total RNA was isolated using the RNeasy kit (Qiagen; Germantown, MD), according to the manufacturer’s instructions. NLRP3 and IL-1β mRNA expressions were measured using qRT-PCR. Total RNA was reverse transcribed using the High-Capacity cDNA Archive Kit (Thermo Fisher Scientific, USA). qRT-PCR was then performed by the coamplification of the NLRP3 gene vs *TBP* (endogenous control) using gene-specific TaqMan Gene Expression Assays using the following primers (Gene Symbol: NLRP3, Hs00918082; assay ID: 2195034G4; *TBP:* Gene Symbol: *TBP*, Hs004227620; Assay ID: 2213015C6; Gene Symbol: IL1B, Hs01555410; Assay ID: 1926685C1 from Thermo Fisher Scientific). Target mRNA expressions were analyzed using the comparative Ct method. Experiments were performed at least three times with three replicates for each experimental group.

### STZ-Induced Mouse Model of Diabetic Retinopathy

Diabetes was induced chemically in 6–8-week-old C57BL/6 mice, according to the method previously described.^36–38^ Briefly, mice were fasted for 4 h and then intraperitoneally injected with 50 mg/kg STZ dissolved in sodium citrate buffer (0.01 M, pH 4.5) on five successive days. Diabetes inductions were monitored 3 days after the last dose of STZ by testing blood glucose levels with a glucometer. Fasting blood glucose levels higher than 300 mg/dL were considered diabetic. The mice were weighed on a weekly basis. Age-matched, nondiabetic C57BL/6 mice were used as healthy controls.

#### Electroretinography (ERG) Assay

Adult 6–8-week-old male and female C57BL/6 mice were injected with or without InflammaProbe-2, dye control, and vehicle control. One week postinjection, the mice were subjected to scotopic ERG according to the previously described protocols.^39^ Animals were dark-adapted overnight, anesthetized with ketamine/xylazine, dilated with 1% tropicamide, and placed on a warm platform within the Ganzfeld dome of a Diagnosys LLC Espion Electrophysiology system (Lowell, MA). Then, the mice were exposed to flashes of light 100 cd.s/m^2^, and the amplitudes of a-wave and b-wave were measured from baseline to peak. The amplitudes of the a-wave and b-wave were plotted as a function of luminance.

### Direct Method for Imaging NLRP3 Inflammasomes in Mouse STZ Retinas

InflammaProbe-2 (10 mg/kg in PBS) was administered to STZ animals by intraperitoneal injection. Four hours postinjection, in vivo InflammaProbe-2-dependent fluorescence imaging was performed according to our previously published method.^40^ Briefly, the mice were anesthetized with ketamine/xylazine and the eyes were dilated with 1% tropicamide and placed on a warm platform; fluorescent and brightfield fundus images were acquired using a Micron IV retinal imaging system (Phoenix Research Laboratories; Pleasanton, CA). After imaging, the animals were sacrificed and enucleated, and the globes were fixed in 10% neutral buffered formalin (NBF). Then, ex vivo imaging of the InflammaProbe-2-dependent fluorescence was performed. In addition, the eyes were prepared for retinal cross sections, stained directly with Alexa Fluor 647-conjugated NLRP3 inflammasome (Cell Signaling Technologies, catalog no. 15101S), and mounted using a microscope slide with a Prolong Gold mounting medium (Life Technologies, Grand Island, NY). Images were captured using an epifluorescence Nikon Eclipse Ti-E inverted microscope (Melville, NY).

### TUNEL Assay

To monitor apoptotic cells in STZ-retinal cross sections, TUNEL assays were performed by using a Click-iT in situ apoptosis detection kit (Life Technology). Adult C57BL/6 mice were treated with STZ to generate the diabetic retinopathy model, as described above. At three months postdiabetes induction, the animals were sacrificed, and the eyes were enucleated. The eyes were fixed in 10% NBF overnight and cross-sectioned (7 μm sections). The retinal cross sections were then stained for fragmented DNA by incorporating an alkyne-modified EdUTP nucleotide, followed by detection with an Alexa Fluor 647 azide in apoptotic cells. Retinal cross sections from a separate group of adult C57BL/6 mice were used as a nondiabetic control. In addition, retinal cross sections from these healthy adult C57BL/6 mice were treated with DNase 1 and stained for TUNEL-positive control assays. These control experimental data are available in Supporting Information Figure S1.

### Cell Proliferation Assay

Cell viability was measured by following our previously published method by using a Calcein Deep Red AM ester (AAT Bioquest, cat# 21902) cell viability assay kit.^29^ Briefly, ARPE-19 cells were cultured in 96-well plates to a 70% confluency in the complete DMEM/F12 (1:1) media with 2% FBS. Before treatments, cells were serum fasted overnight. The next day, the cells were treated with 100 μL of media containing different concentrations (0–100 μM) of either InflammaProbe-2 or MCC950 and incubated for 24 h. Seventy percent ethanol was used as a negative control for cell viability and cultured on a separate 96-well plate. All conditions had 6 replicates. After 24 h, the media were removed and replaced with fresh 0% FBS media containing a 5 μM Calcein Deep Red AM ester, a nonfluorescent compound that is cleaved into a brightly fluorescent product (λ_ex_/λ_em_ = 643/663 nm) by esterases within live cells. This fluorescent compound is red-shifted relative to InflammaProbe-2 (λ_ex_/λ_em_ = 490/530 nm), which was necessary to avoid interference from any InflammaProbe-2-dependent fluorescence during fluorometric measurements. The cells were incubated at 37 °C for 1 h. Then, the media were removed, and 100 μL of prewarmed Hank’s Balanced Salt Solution (Gibco, catalog no. 14025092) was added to the wells. Fluorescence was measured at λ_ex_/λ_em_ = 620/660 nm using the Cytation 5 microplate reader (Agilent BioTek, Santa Clara, CA). Fluorescence intensities were plotted as the percentage of cell viability relative to the control group. The data were expressed as the mean ± SD of six replicates per group.

## Statistics

For data presentation, mean ± SD was used. To compare two sample types, Student’s tests were performed. In addition, to compare more than two sample types, one-way ANOVA, followed by Dunnett’s multiple comparison test, was performed using Prism 10 software (GraphPad, San Diego, CA). To monitor statistical significance, a *P*-value of ≤0.05 was considered statistically significant.

### Synthesis of InflammaProbe-2

Schematic presentation for chemical synthesis of InfmammapProbe-2 and related compounds has been included in the Supporting Information (SI). Also, detailed methods for the synthesis and characterization data for InflammaProbe-2 and related compounds are provided in the Supporting Information. The HPLC method was used to determine the purity of the InflammaProbe-2 compound, confirming >99% purity as shown in the Supporting Information.

## Supplementary Material





## Data Availability

All data that support the results of this study are available within the article and its Supporting Information or upon request to the corresponding author (MIU).

## Notes

General chemistry techniques; chromatography (Figure S1); the TUNEL assays (Figure S2); molecular docking of InflammaProbe-2 (Figure S3); perinuclear staining of InflammaProbe-2 (Figure S4); time-dependent increase of InflammaProbe-2 fluorescence (Figure S5); inflammaProbe-2 concentrations in whole blood over time (Figure S6); stability of InflammaProbe-2 in blood serum over time (Scheme 1); multistep synthesis of InflammaProbe-2; synthesis of Compound 3; synthesis of Compound 2; synthesis of InflammaProbe-2 free base; synthesis of InflammaProbe-2 (Na-salt) (Scheme 2); chemical synthesis of Compound 4 and Compound 1; synthesis of Compound 4; synthesis of Compound 1 (Table S1); microscopic configuration of the TUNEL assay (microscopy and image processing details pertaining to Figures 4 and 11); microscopic configuration for in vitro imaging (microscopy and image processing details pertaining to Figure 5) (Table S2); microscopic configuration for in vitro imaging (microscopy and image processing details pertaining to Figure 6) (Table S3); microscopic configuration for ex vivo imaging (microscopy and image processing details pertaining to Figure 10) (Table S4); microscopic configuration for in vitro imaging (microscopy and image processing details pertaining to Figures 7, 8 and S4) (Table S5) (PDF)
Molecular formula string (CSV)

## Abbreviations Used

DAMPs: danger-associated molecular patterns
DR: diabetic retinopathy
ERG: electroretinography
FA: fluorescein angiography
NLRP3: NLR-family pyrin domain-containing 3
NPDR: nonproliferative diabetic retinopathy
NV: neovascularization
OCT: optical coherence tomography
PAMPs: pathogen-associated molecular patterns
PDR: proliferative diabetic retinopathy
VEGF: vascular endothelial growth factor
