# Supplementary material for: Novel Optical Imaging Probe for the Targeted Visualization of NLRP3 Inflammasomes in Living Retina
Source: J Med Chem. 2025 Aug 4;68(15):16034–47. doi: 10.1021/acs.jmedchem.5c00999 (PMC12362592; doi:10.1021/acs.jmedchem.5c00999)
Supplement: Supplementary file 1 [file jm5c00999_si_001.pdf]

# Supporting Information

# Novel Optical Imaging Probe for the Targeted Visualization of NLRP3 Inflammasomes in living retina

MD Imam Uddin,*[1,2] Blake Dieckmann,[1] Sarah Soliman[1]

**Authors' Affiliations**

[1]Department of Ophthalmology and Visual Sciences, Vanderbilt University School of Medicine, Nashville, TN 37232, USA.

[2]Department of Biomedical Engineering, Vanderbilt University School of Engineering, Nashville, TN 37235, USA.

***Corresponding author email address:**

MD Imam Uddin, PhD

E-mail: md.i.uddin@vumc.org

## Contents of Supporting Information

General Chemistry Techniques ----------------------------------------------------------------------page S3

Chromatography ---------------------------------------------------------------------------------------------S3

Figure S1: The TUNEL assays ---------------------------------------------------------------------------S4

Figure S2: Molecular docking of InflammaProbe-2 ----------------------------------------------------S5

Figure S3: Perinuclear staining of InflammaProbe-2 -------------------------------------------------S6

Figure S4: Time dependent increase of InflammaProbe-2 fluorescence ----------------------------S7

Figure S5: InflammaProbe-2 concentrations in whole blood over time -----------------------------S8

Figure S6: Stability of InflammaProbe-2 in blood serum over time ---------------------------------S9

Scheme 1: Multi-step synthesis of InflammaProbe-2 -----------------------------------------------S10

Synthesis of Compound 3 ----------------------------------------------------------------------------------S11
Synthesis of Compound-2 ----------------------------------------------------------------------------------S13
Synthesis of InflammaProbe-2 free base --------------------------------------------------------------------S15
Synthesis of InflammaProbe-2 (Na-salt) --------------------------------------------------------------------S17
Scheme 2: Chemical synthesis of Compound-4 and Compound-1 ---------------------------------S20
Synthesis of Compound 4 ----------------------------------------------------------------------------------S20
Synthesis of Compound 1 ----------------------------------------------------------------------------------S22
Table S1: Microscope configuration for TUNEL assay ------------------------------------------------S25
(Microscopy and image processing details pertaining to Figure 4 and Figure 11)
Table S2: Microscope configuration for in vitro imaging ------------------------------------------------S26
(Microscopy and image processing details pertaining to Figure 5)
Table S3: Microscope configuration for in vitro imaging ------------------------------------------------S27
(Microscopy and image processing details pertaining to Figure 6)
Table S4: Microscope configuration for ex vivo imaging -----------------------------------------------S28
(Microscopy and image processing details pertaining to Figure 10)
Table S5: Microscope configuration for in vitro imaging ------------------------------------------------S29
(Microscopy and image processing details pertaining to Figure 7, 8 and Figure S4)

## General Chemistry Techniques

All chemicals were purchased and used as received unless otherwise indicated. Moisture sensitive reactions were performed in oven-dried glassware under a positive pressure of argon. Air and moisture-sensitive compounds were introduced via syringe or cannula through a rubber septum. HPLC grade solvents were obtained from Fisher Scientific (Pittsburgh, PA). All reagents and deuterated solvents were purchased from the Aldrich Chemical Company (Milwaukee, WI) and used without further purification. The Oregon Green 488 carboxylic acid succinimidyl ester, 5-isomer was purchased from Life Technologies (Grand Island, NY) and used without further purification.

## Chromatography

Silica gel column chromatography was performed using Sorbent silica gel standard grade, porosity 60 Å, particle size 32-63 (μm) (230 x 450 mesh), surface area 500-600 m2/g, bulk density 0.4 g/mL, pH range 6.5-7.5, purchased from Sorbent Technologies (Atlanta, GA). The analytical HPLC of the fluorescent compounds were performed on a Waters 2996 HPLC system with a UV or fluorescence detector using C18 reverse-phase columns. InflammaProbe-2 and related compounds used for biological assays were ≥ 95% purity based on analytical HPLC analysis.

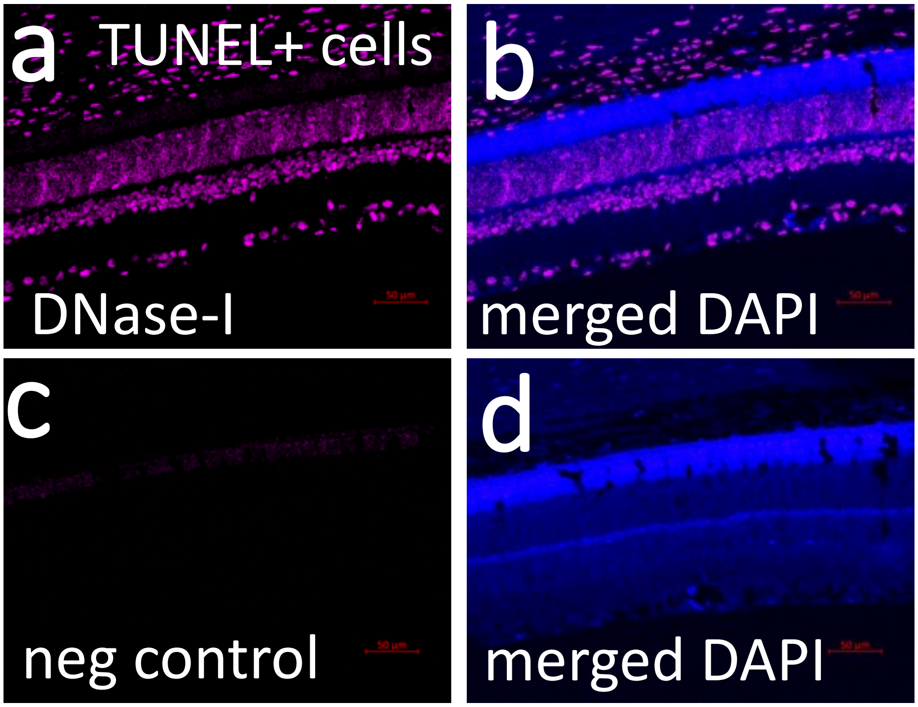


**Figure S1:** The TUNEL assays were performed in retinal cross sections to assess retinal cell apoptosis. (a, b) DNase-1 treated retinal cross sections were used as positive control, fragmented DNA was clearly visible; (c, d) Healthy control retinal cross sections were used negative control showing no cellular apoptosis. Scale bar in images a-d, 50 µm.

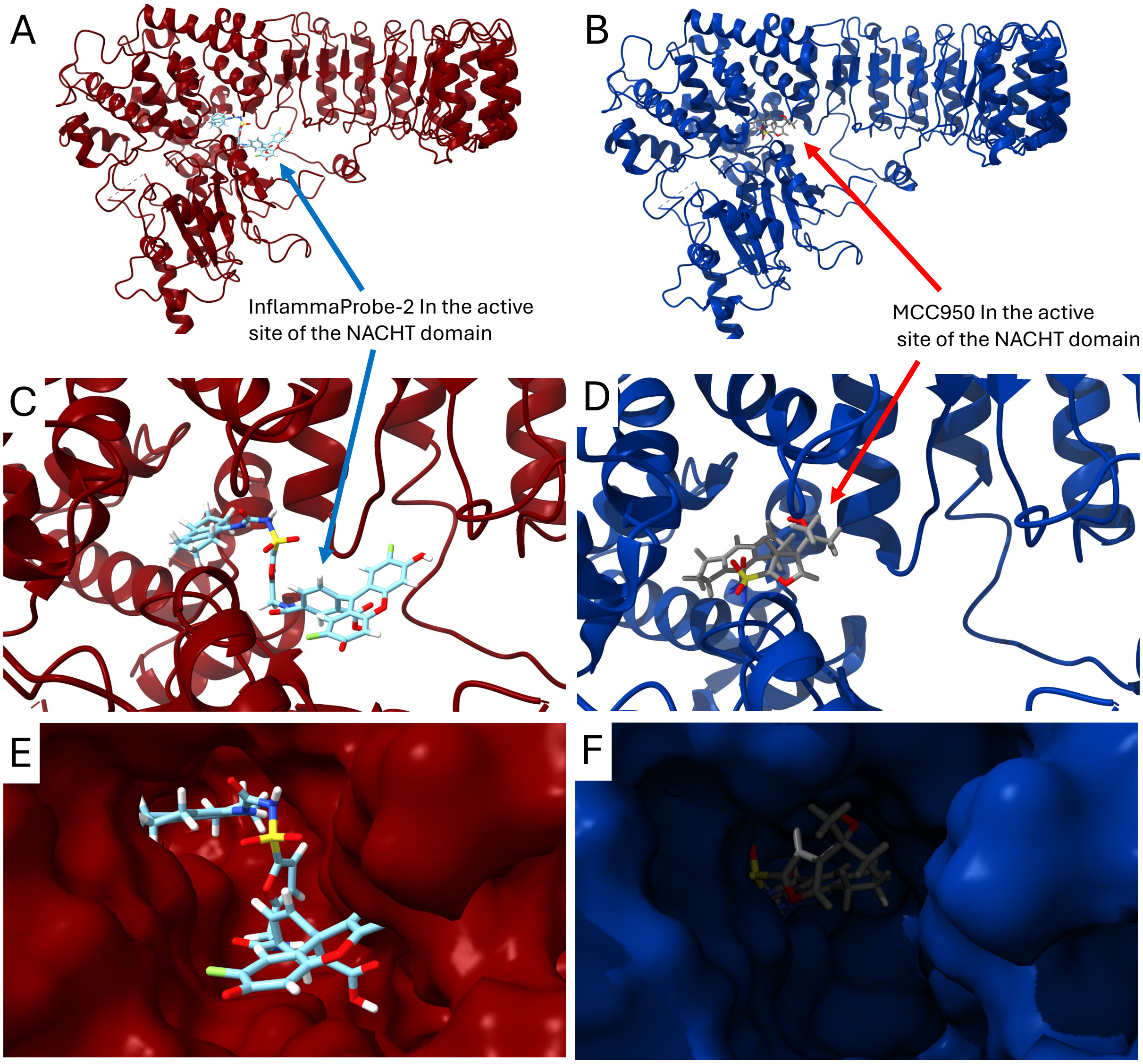


**Figure S2:** Molecular docking of InflammaProbe-2; AutoDock program was used for the molecular modeling using PDB ID: 7PZC. (A) Based on lowest energy conformation, InflammaProbe-2 binds to the active site of the NACHT domain. (B) Cryo-EM structure of NACHT domain with MCC950 showing MCC950 at active site of the NACHT domain. (C, D) Magnification view of A and B respectively showing the ligands binding at the active site of the NACHT domain. (E) Surface view of the modeling of InflammaProbe-2 in the active site of the pocket. Both MCC950 and InflammaProbe-2 shares similar binding patterns at the active site of the NLRP3 inflammasome.

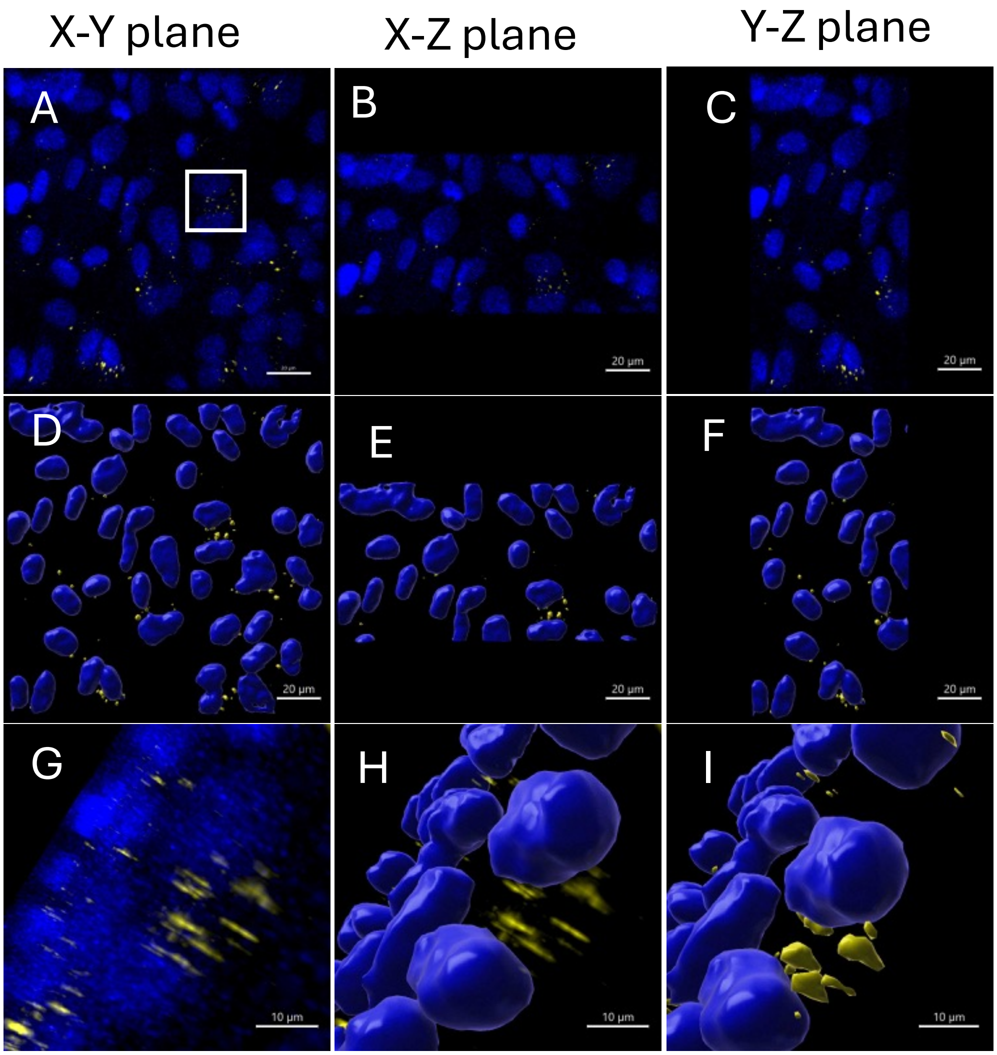


**Figure S3:** Perinuclear staining of InflammaProbe-2. ARPE-19 cells were treated for 3 hours with hyperglycemia and InflammaProbe-2. (A-C) View of maximum intensity projections in the X-Y, X-Z, and Y-Z planes showing InflammaProbe-2 fluorescence (yellow) is mainly found near but outside of the nucleus (perinuclear) sites of the ARPE-19 cells. The nucleus was stained with DAPI (blue). (D-F) Shows a 3-D rendering of (A-C). (G-H) Corresponds to area in the white rectangle shown in (A) and has been visualized on its axis for clear view of the perinuclear location of InflammaProbe-2. All 3-D renderings were created using IMARIS software.

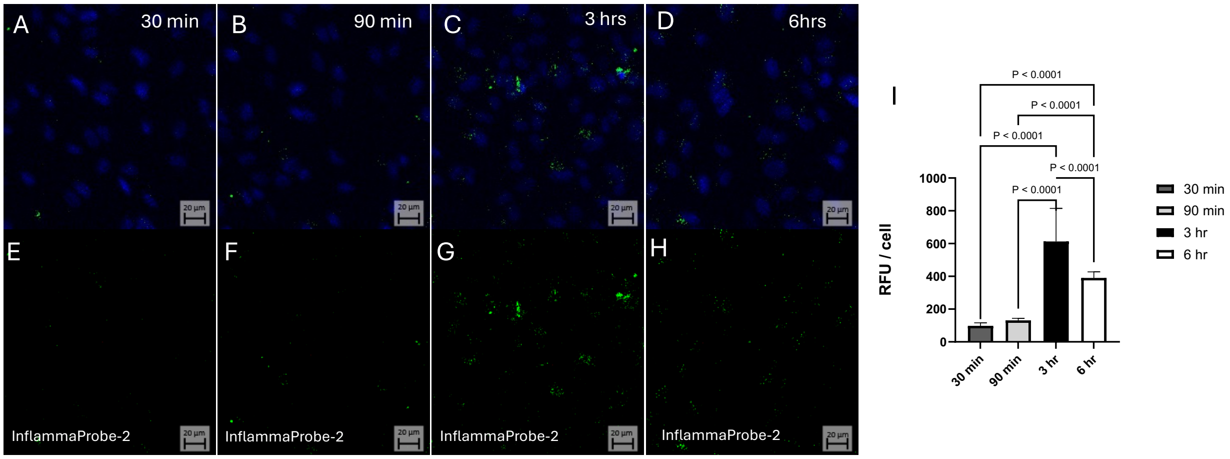


**Figure S4:** Time dependent increase of InflammaProbe-2 fluorescence intensities at 50 mM glucose treatment condition. ARPE-19 cells were incubated in media with high glucose concentration (50 mM) and the InflammaProbe-2 fluorescence was monitored over time. Cells were incubated at each timepoint with inflammaprobe-2 for three hours. Therefore, at the 30 minute timepoint, cells were preincubated with inflammaprobe-2 for two and half hours in normoglycemia before being incubated with 50 mM glucose and InflammaProbe-2 (10 $\mu$M) for 30 minutes. (A, E) At 30 minutes timepoint InflammaProbe-2 fluorescence was minimally detected, suggesting minimal levels of NLRP3 inflammasomes activation at this timepoint. (B, F) At 90 minutes timepoint, slight increase in InflammaProbe-2 fluorescence was detected compared to 30 minutes timepoint. (C-D, G-H) The inflammaprobe-2 signal has further increased significantly at the 3 and 6 hours timepoints compared to the 30 minutes and 90 minutes timepoints. However, the fluorescence signal decreased significantly at 6 hours compared to 3 hours (p = <0.0001). These data indicate that the activation of NLRP3 inflammasome requires time at the early stage to initiate the activation. After the activation, the activated NLRP3 inflammasomes may initiate programmed cell death which may reflects at the 6 hours timepoint. These are representative images from n = 3 replicates.

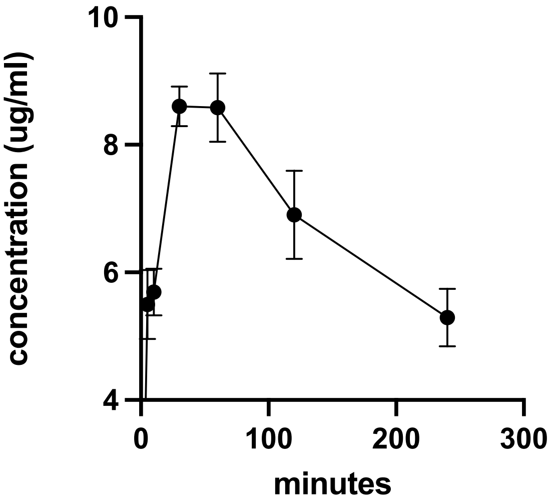


**Figure S5:** InflammaProbe-2 concentrations in whole blood over time. Mice were injected with InflammaProbe-2 (10 mg/kg). Blood samples were analyzed using plate-based assay to quantify the amount of InflammaProbe-2 in the blood circulation at different timepoint. For each time point, blood samples from three animals were analyzed. The data indicate that InflammaProbe-2 is almost cleared from blood circulation after three hours.

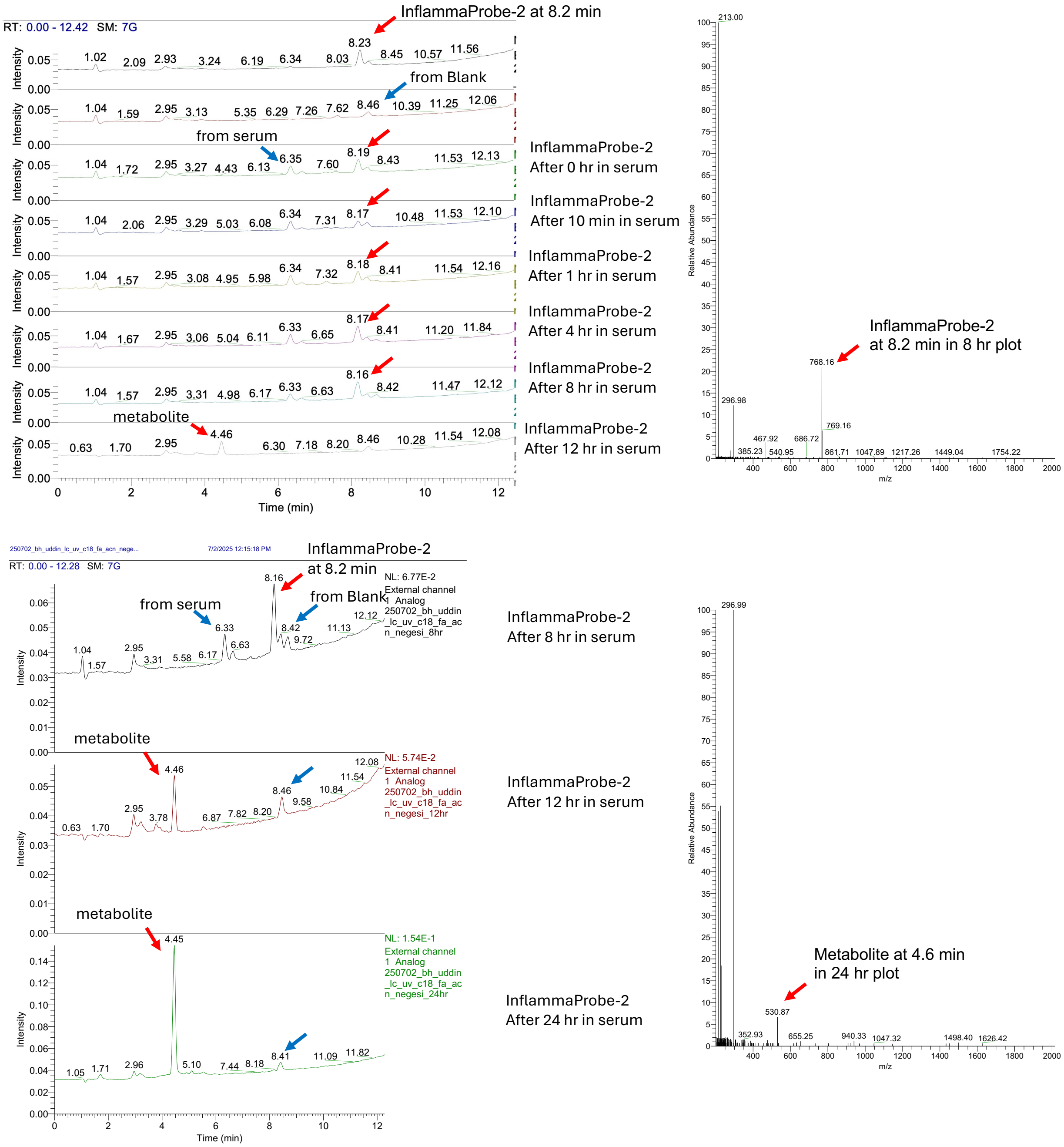


**Figure S6:** Stability of InflammaProbe-2 in blood serum over time. Blood was collected from C57BL/6 WT mice at room temperature; centrifuged and serum was isolated. InflammaProbe-2 was added to the serum to give a final concentration of 350 μM in blood serum medium and incubated at 37 °C. At given timepoints (10 min, 1 hr, 8 hr, 12 hr, 24 hr) 100 μL of serum was pulled from the vial and extracted in 250 μL ethyl acetate with 0.5% formic acid. Mixture was vortexed, layers were allowed to separate, and ethyl acetate layer was moved to a new tube. Ethyl acetate was removed via a stream of argon air and sample was frozen at -80 degrees until

processing using HPLC system. Only serum and ethyl acetate were used as Blank. At given timepoints, 100 μL of InflammaProbe-2-serum mixture was pulled and processed as describe above. We observed a metabolite from InflammaProbe-2 in serum medium after 12 hours in serum. Almost entire InflammaProbe-2 remained as intact probe after 8 hours incubation in serum medium at 37 °C. The LCMS data showed the metabolite has a MS of 530.87 and 296.99.

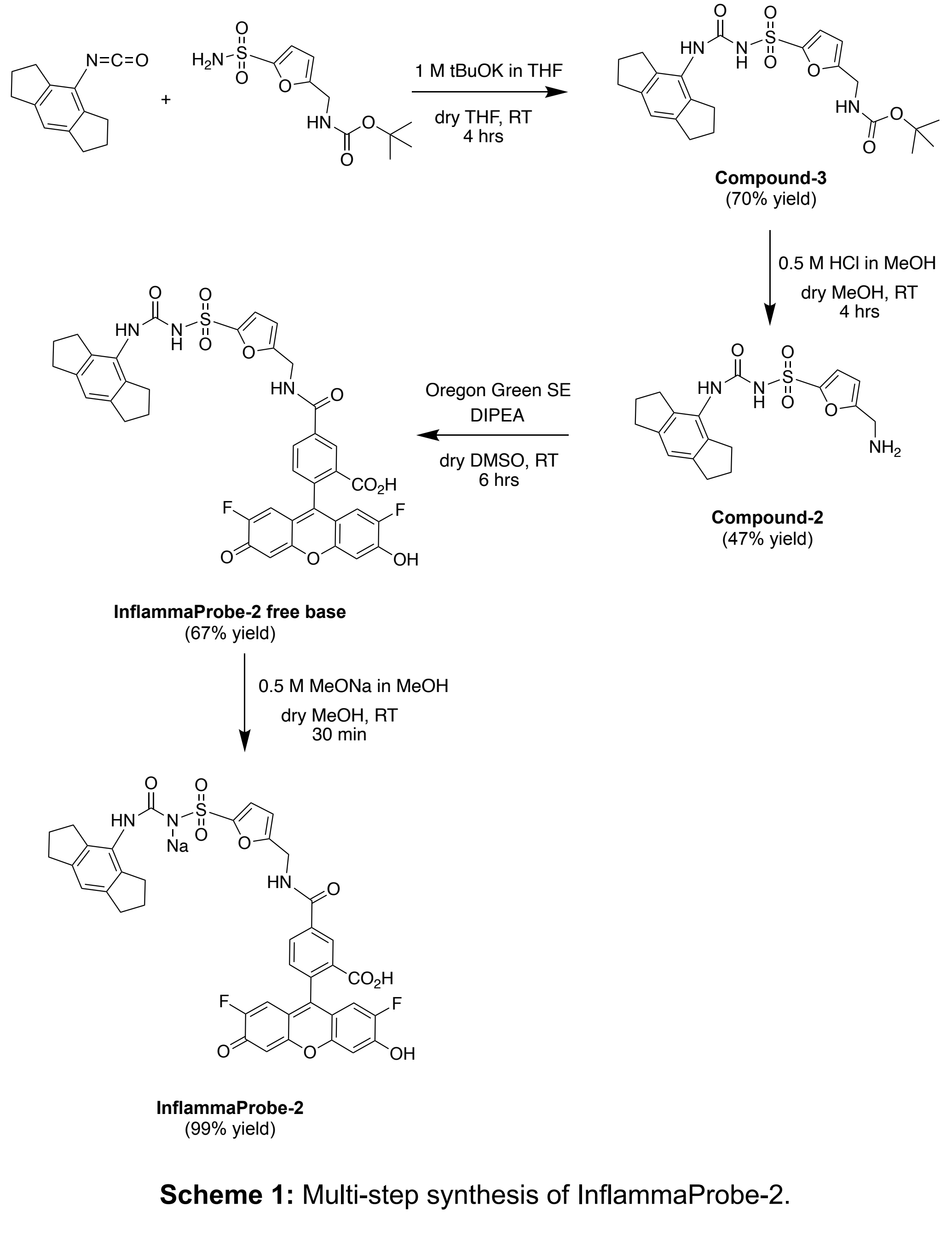


**Scheme 1:** Multi-step synthesis of InflammaProbe-2.

## Synthesis of Compound 3

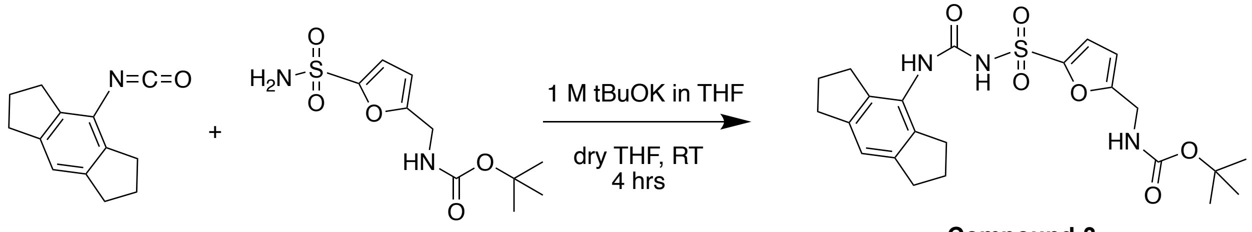


**Compound-3**
(70% yield)

InflammaProbe-2 was synthesized in multi-step reactions starting from the synthesis of Compound-3. **Compound-3** was synthesized by the following procedure: *tert*-Butyl ((5-sulfamoylfuran-2-yl)methyl)carbamate (276 mg, 1.00 mmol) was dissolved in dry THF (6 mL). To this solution, tBuOK (1M in dry THF, 1.5 mL, 1.50 mmol) was added at 0 °C under argon and the reaction mixture was stirred at 0 °C for 5 min. Then 4-isocyanato-1,2,3,5,6,7-hexahydro-*s*-indacene (200 mg, 1.00 mmol) was dissolved in dry THF (2 mL) and added to the reaction mixture at 0 °C. The reaction mixture was stirred for 4 hrs at room temperature under argon atmosphere. The organic solvent was removed under reduced pressure. The crude product was purified by silica column chromatography using chloroform methanol mixtures as eluent to yield Compound-3 as a white solid (325 mg).

Yield 70%; 1H NMR (600 MHz, DMSO-d6) δ 1.37 (s, 9H), 1.91 (q, J = 7.4 Hz, 4H), 2.63 (t, J = 7.4 Hz, 4H), 2.75 (t, J = 7.4 Hz, 4H), 4.10 (d, J = 5.8 Hz, 2H), 6.20 (bs, 1H), 6.65 (bs, 1H, NH), 6.81 (bs, 1H), 7.08 (bs, 1H, NH), 7.38 (m, 1H), 7.68 (bs, 1H, NH). HRMS (ESI) m/z [M-H]+ calculated for C23H28N3O6S 474.1699, found 474.1685.

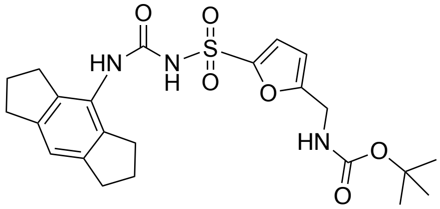

**Compound-3**

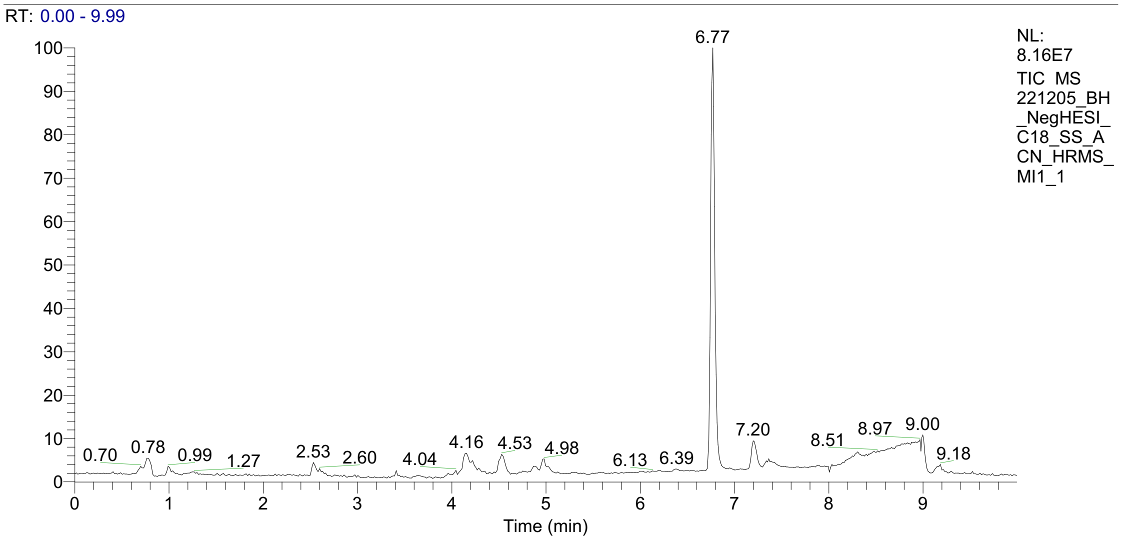


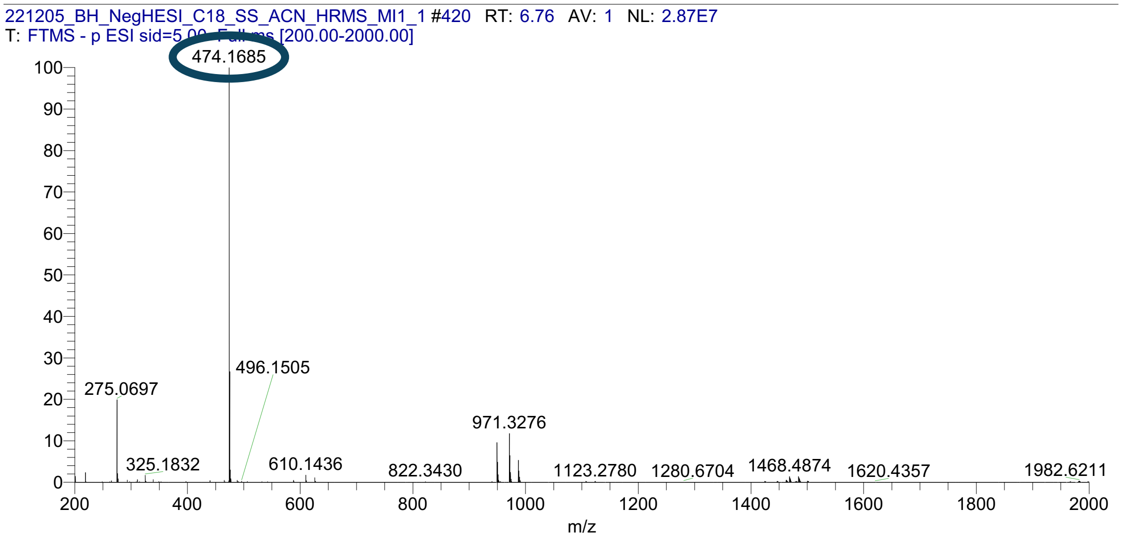


HR-MS spectra of compound-3.

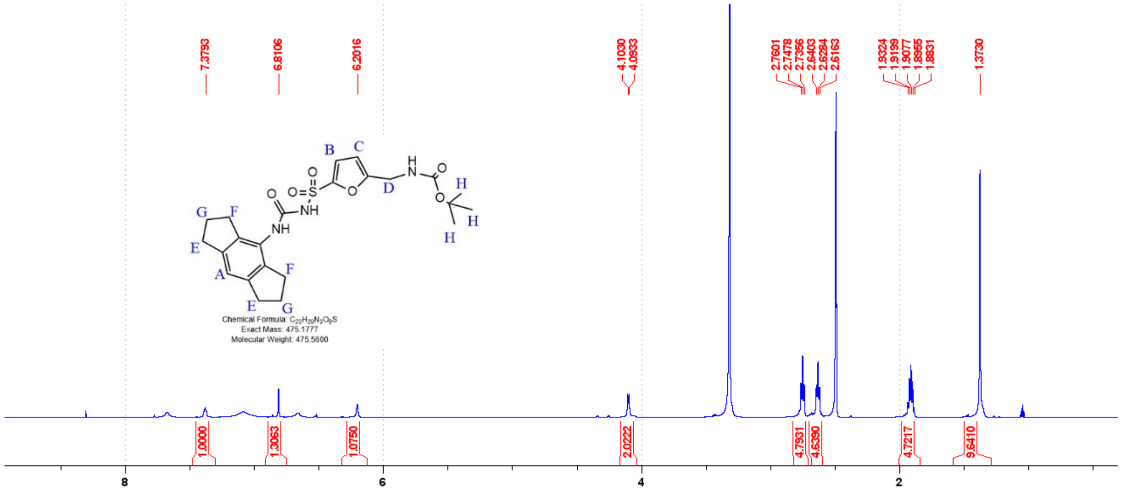


1H-NMR spectrum of Compound-3.

## Synthesis of Compound-2

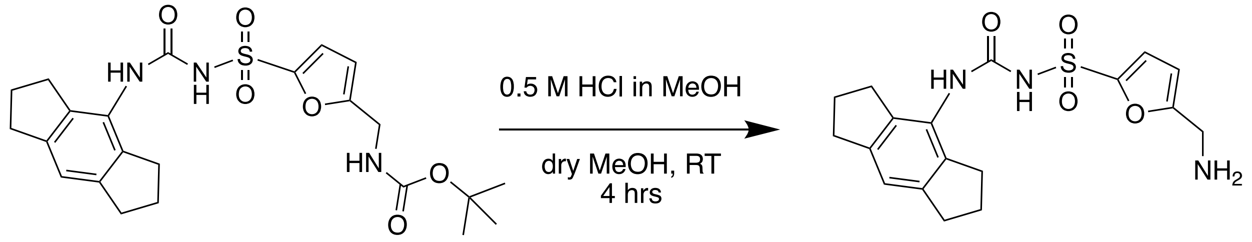


**Compound-3**

**Compound-2**
(47% yield)

**Compound-3** (166 mg, 0.35 mmol) was dissolved in dry MeOH (2 mL). To this solution, HCl (0.5 M in MeOH, 2.0 mL, 1.00 mmol) was added at 0 °C under argon and the reaction mixture was stirred at room temperature for 4 hrs under argon atmosphere. The organic solvent was removed under reduced pressure. The crude product was purified by silica column chromatography using chloroform methanol mixtures as eluent to yield **Compound-2** as a light-brown solid (62 mg).

Yield 47%; 1H NMR (600 MHz, DMSO-d6) δ 1.91 (m, 4H), 2.67 (t, J = 7.4 Hz, 4H), 2.76 (t, J = 7.4 Hz, 4H), 4.11 (s, 2H), 6.51 (d, J = 3.0 Hz, 1H), 6.63 (d, J = 3.0 Hz, 1H), 7.40 (bs, 4H, NH). HRMS (ESI) m/z [M-H]+ calculated for C18H20N3O4S 374.1253, found 374.1180.

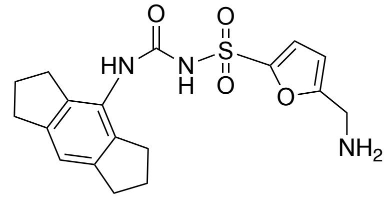


**Compound-2**

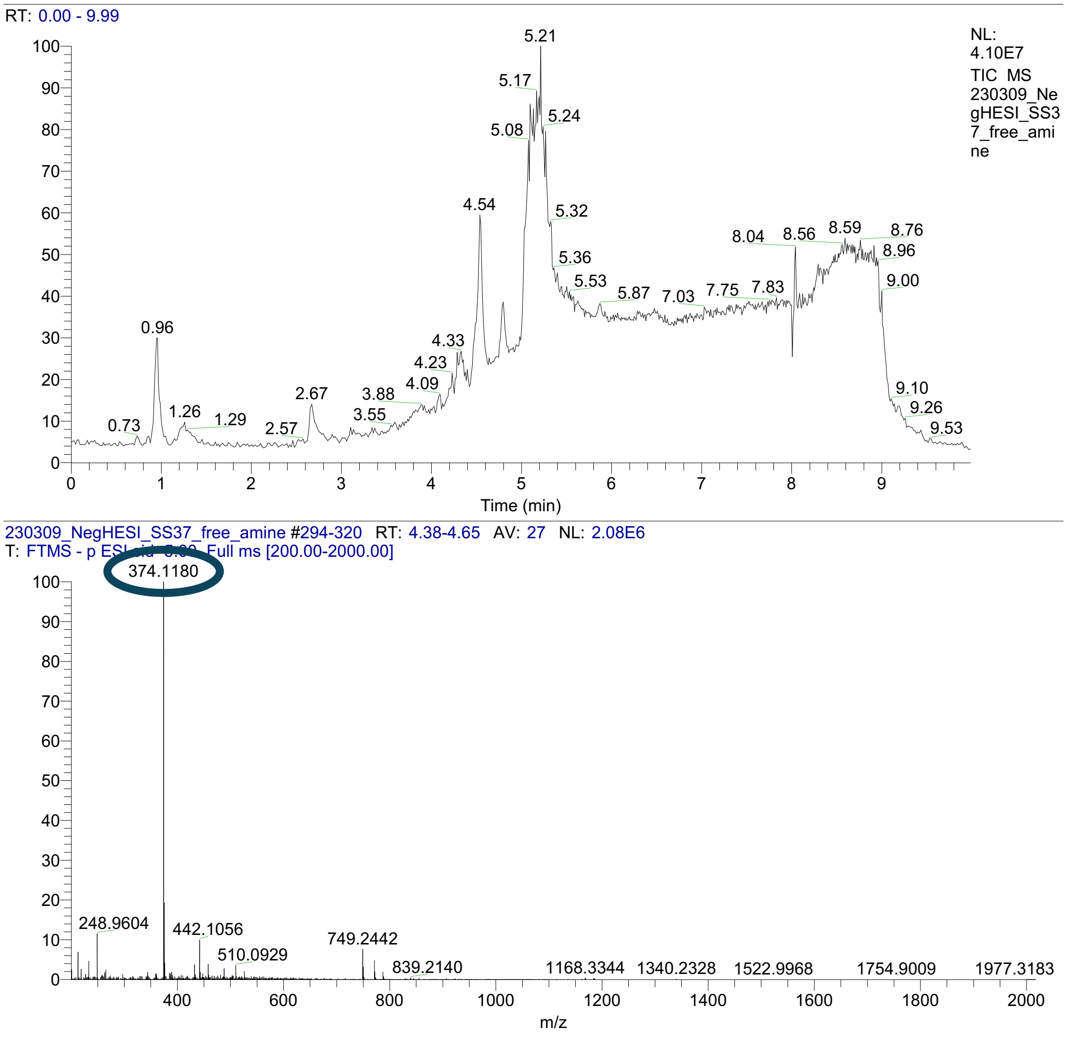


HRMS spectra of compound-2.

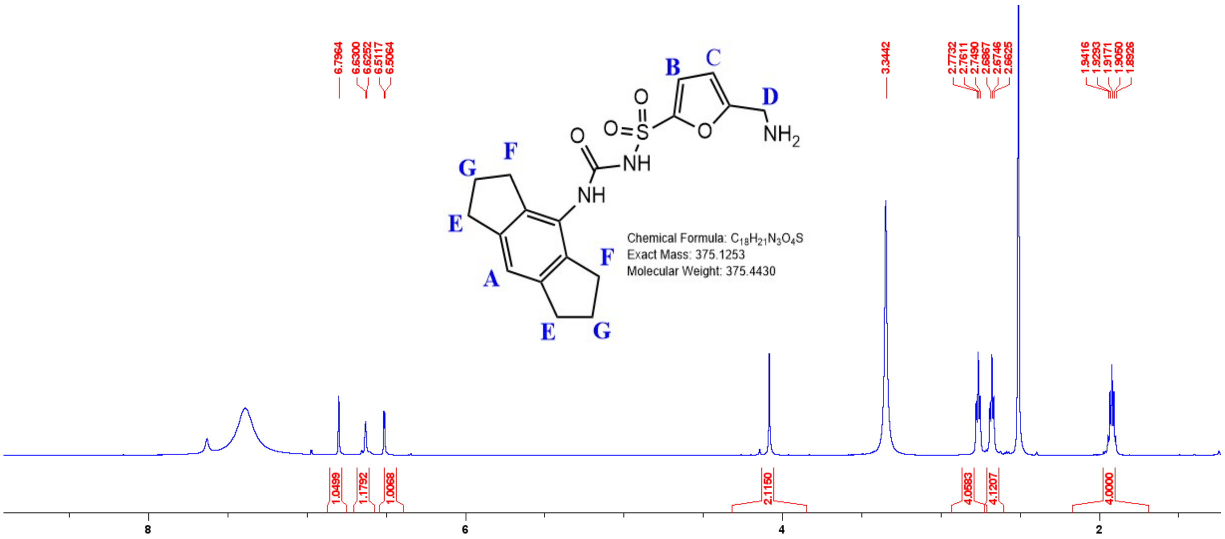


1H-NMR spectrum of Compound-2.

## Synthesis of InflammaProbe-2 free base

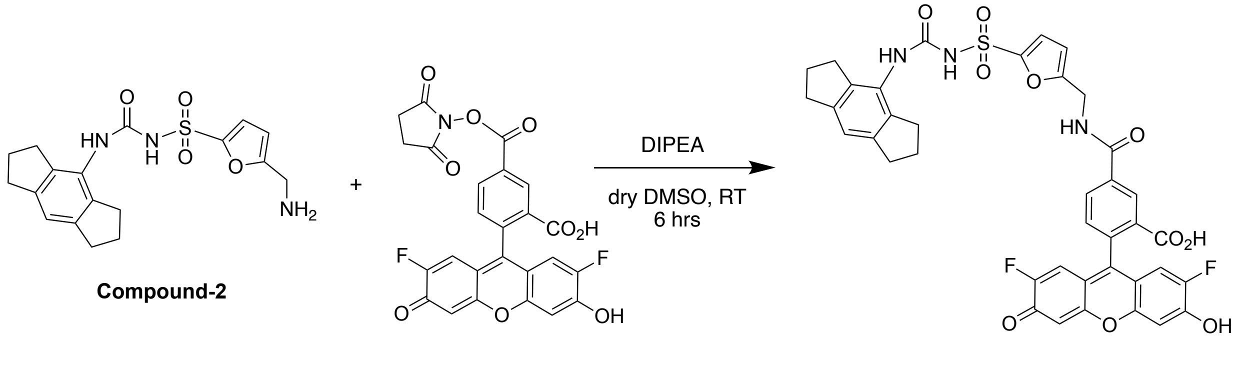


To a stirred solution of **Compound-2**, (23 mg, 61.3 μmol) in dry dimethylsulfoxide (2 mL) N,N-diisopropylethylamine (DIPEA) was added (0.1 μmol) to activate the free amine. After stirring for 5 min, the Oregon Green 5-carboxylic acid succinimidyl ester, (20 mg, 39.3 μmol) was added and stirred overnight at 25 °C. The solvent was removed by lyophyllization to give the crude product, which was purified using HPLC chromatography system using acetonitrile water mixtures as eluent to yield InflammaProbe-2 free base as Orange solid (18 mg).

Orange solid, Yield 67%; 1H NMR (600 MHz, DMSO-d6) δ 1.84 (m, 4H), 2.63 (t, J = 7.4 Hz, 4H), 2.71 (t, J = 7.4 Hz, 4H), 4.50 (d, J = 5.6 Hz, 2H), 6.29 (d, J = 3.2 Hz, 1H), 6.42 (bs, 2H), 6.53 (d, J = 3.2 Hz, 1H), 6.75 (s, 1H), 7.31 (m, 2H), 7.59 (bs, 1H), 8.13 (d, J = 7.9 Hz, 1H), 8.53 (s, 1H), 9.35 (m, 1H). Mass (ESI+) m/z calculated for C39H29F2N3O10S [M+H]: 770.2; found: 770.5. The liquid chromatography method was used to determine the purity confirming >99% purity.

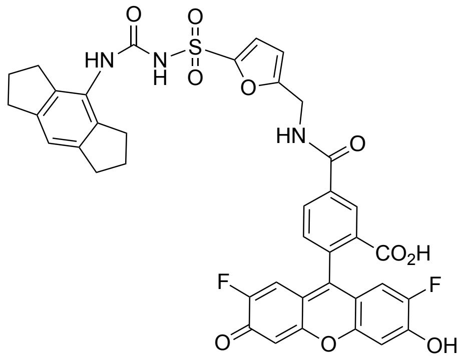

**InflammaProbe-2 free base**

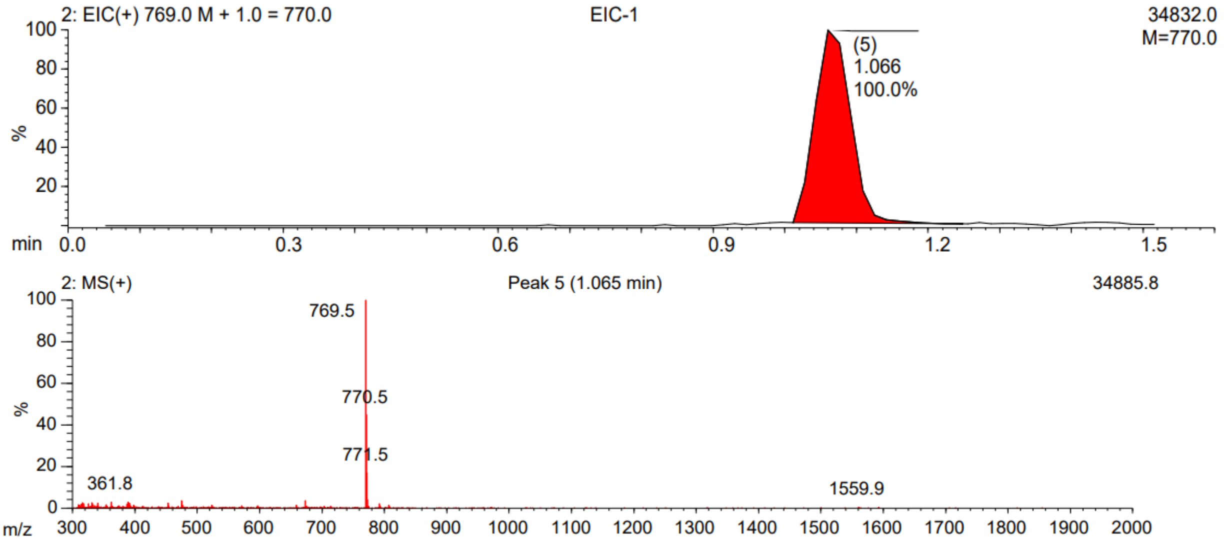


LRMS spectra of InflammaProbe-2 Free base.

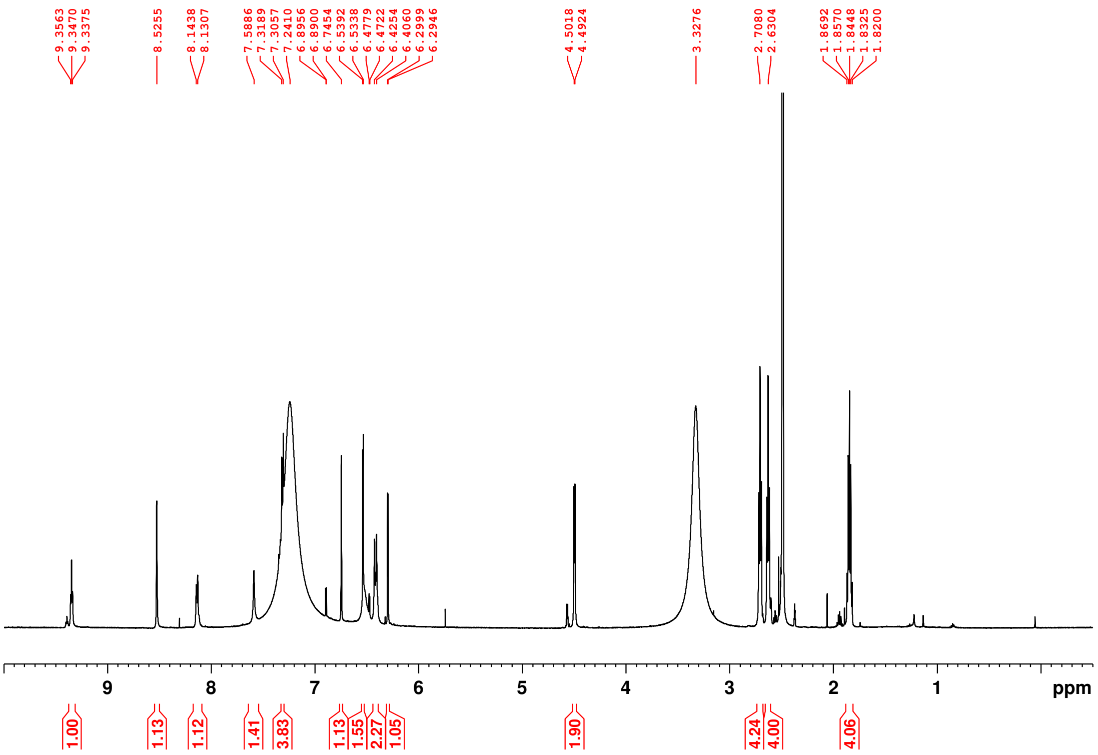


1H-NMR spectrum of InflammaProbe-2 Free base.

## Synthesis of InflammaProbe-2 (Na-salt)

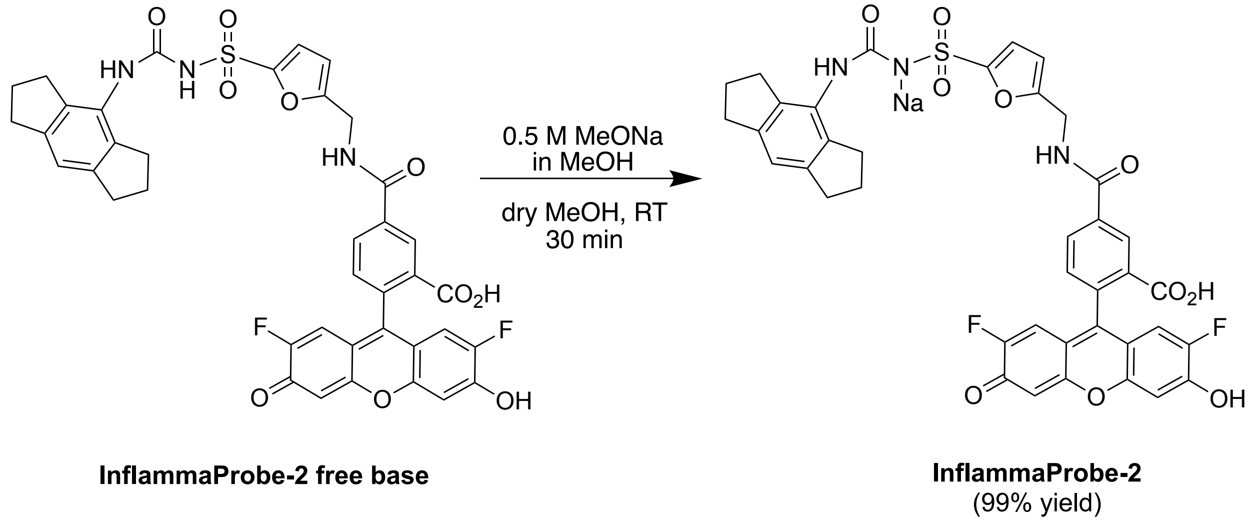


**Method:** InflammaProbe-2 free base (50 mg, 0.064 mmol) was dissolved in dry THF (10 mL). To this solution, NaOMe (0.5 M in MeOH, 2.0 mL, 1.00 mmol) was added at 0 °C under argon

and the reaction mixture was stirred at room temperature for 30 minutes under argon atmosphere. The organic solvent was removed under reduced pressure. The crude product was purified by silica column chromatography using dichloromethane-methanol mixtures as eluent to yield the final compound InflammaProbe-2 as deep orange solid (51 mg).

Yield 99%; 1H NMR (600 MHz, DMSO-d6) δ 1.79 (m, 4H), 2.58 (t, J = 7.4 Hz, 4H), 2. 56 (t, J = 7.4 Hz, 4H), 4.43 (d, J = 5.6 Hz, 2H), 6.22 (d, J = 3.2 Hz, 1H), 6.31 (m, 2H), 6.44 (d, J = 3.2 Hz, 1H), 6.67 (s, 1H), 7.25 (m, 2H), 7.46 (s, 1H), 8.05 (m, 1H), 8.46 (d, J = 1.3 Hz, 1H), 9.27 (m, 1H). 19F-NMR (500 MHz, DMSO-*d6*) δ -73.44. HRMS (ESI) m/z [M-Na]+ calculated for C39H28F2N3O10S 768.1464, found [M-Na] 768.1511.

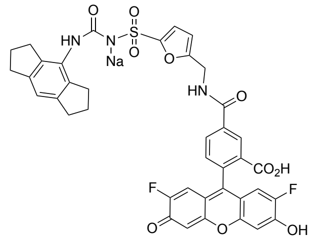


**InflammaProbe-2**

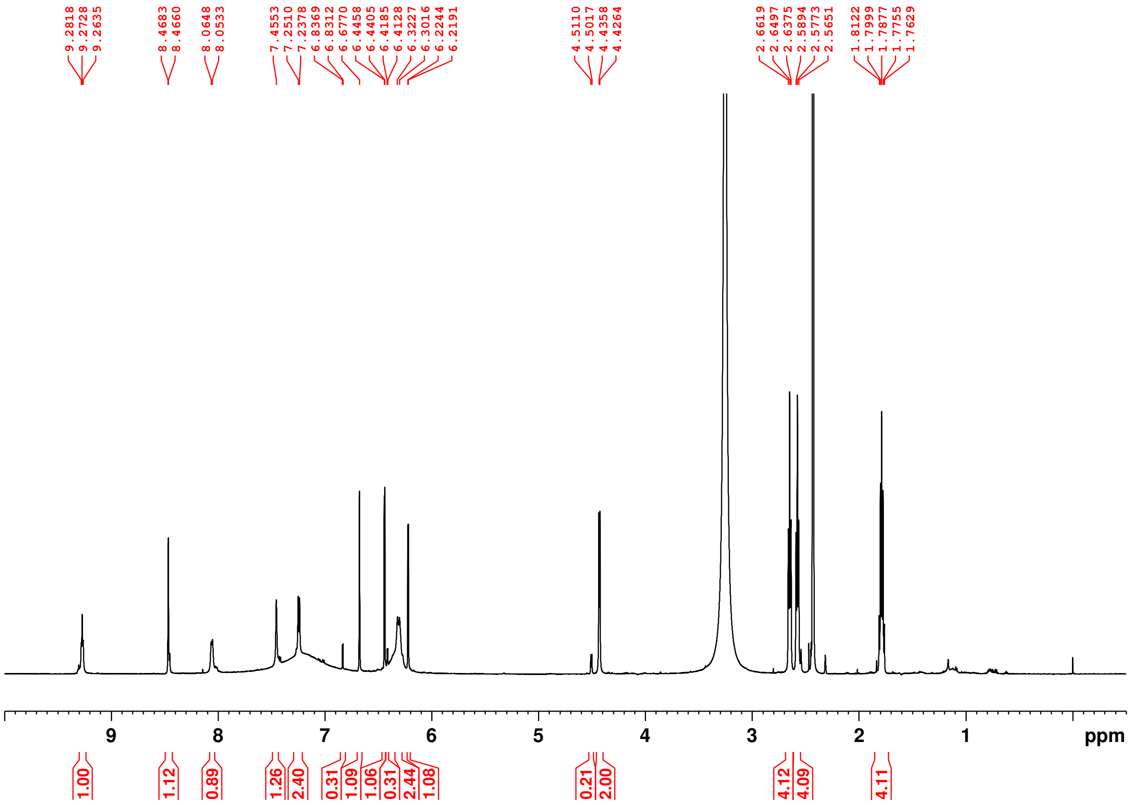


1H-NMR spectrum of InflammaProbe-2.

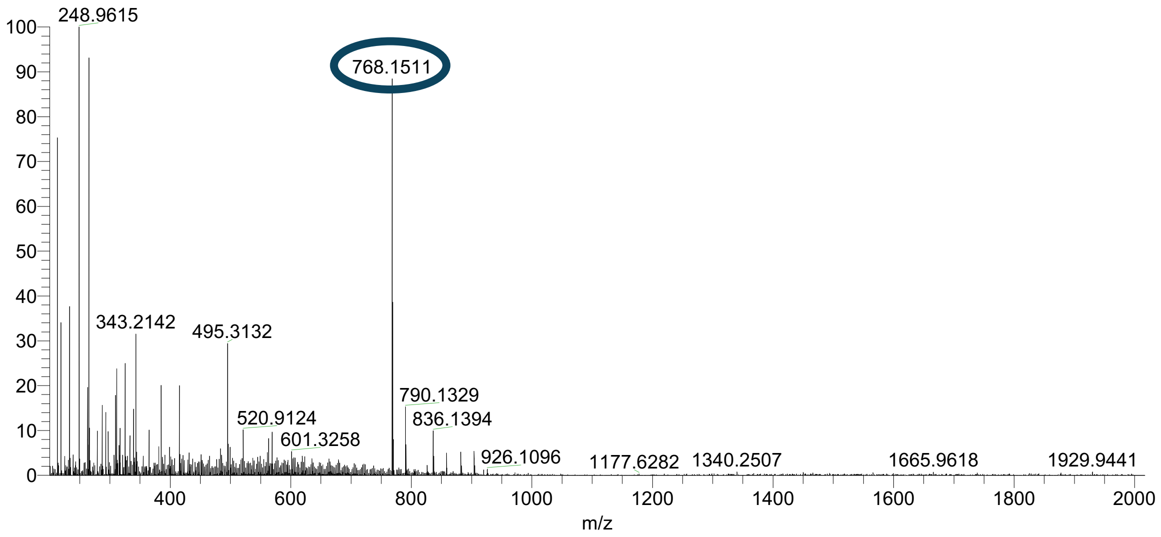


HR-MS spectra of InflammaProbe-2.

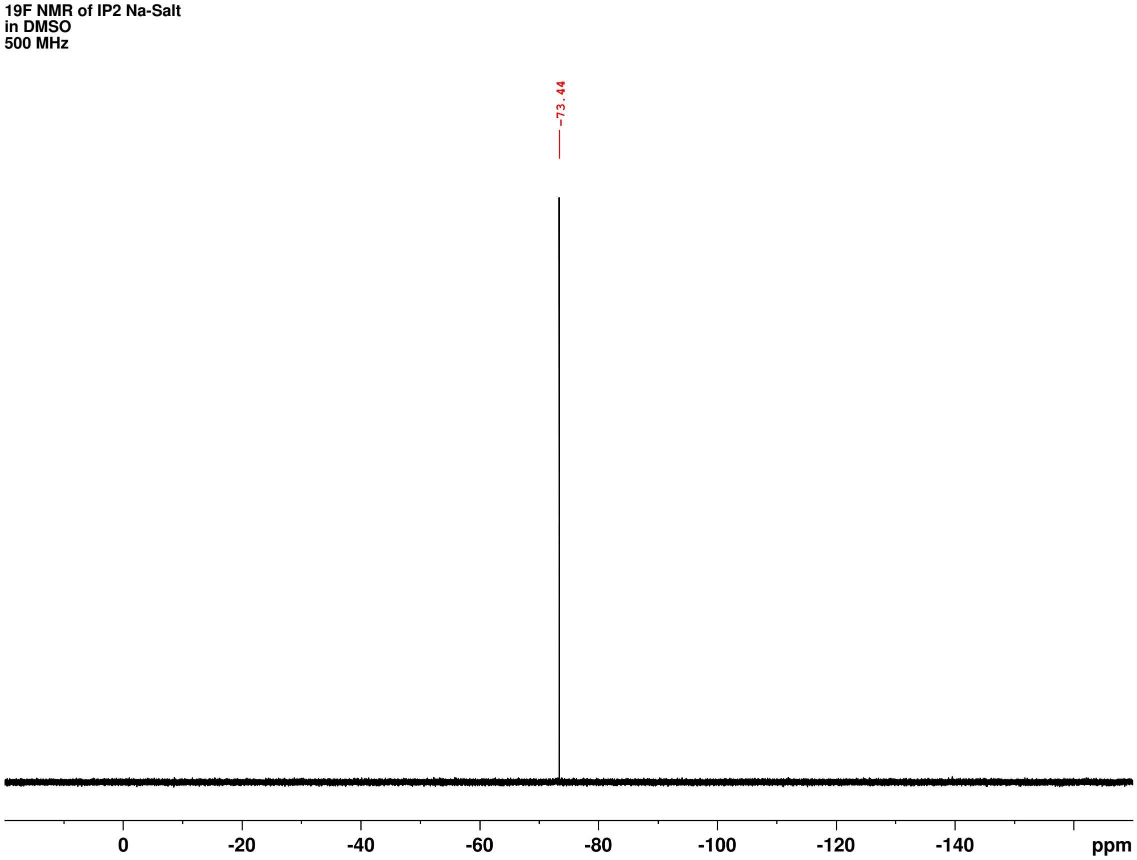


19F-NMR spectrum of InflammaProbe-2.

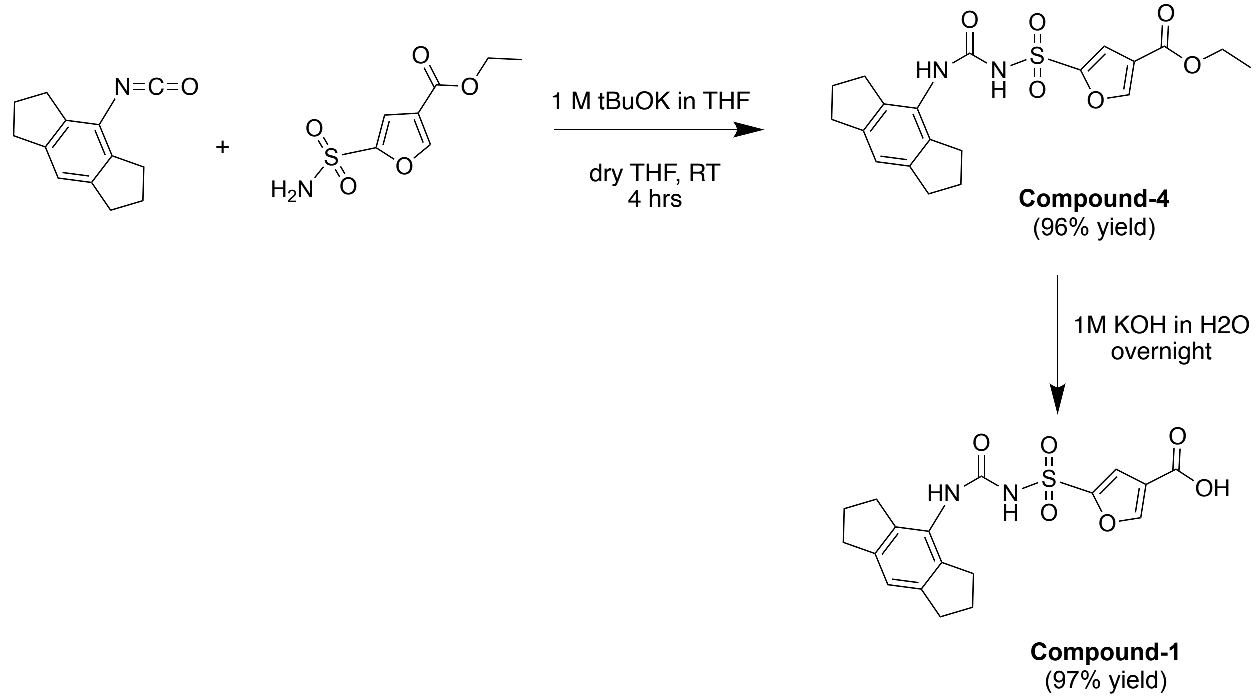


***Scheme 2:*** Chemical synthesis of **Compound-4** and **Compound-1**.

### Synthesis of Compound-4

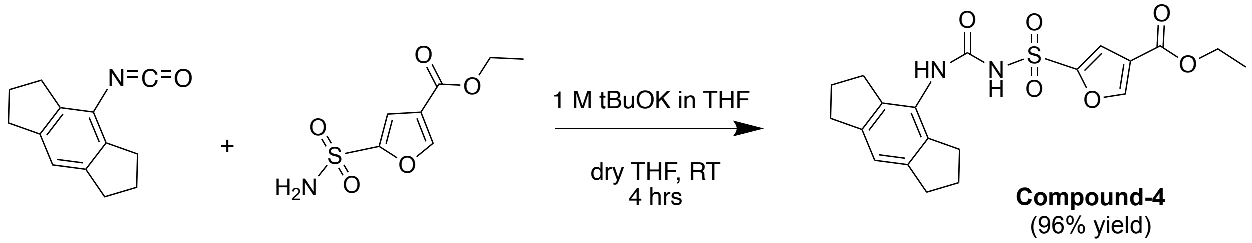


**Compound-4** was synthesized by the following procedure: *tert*-Butyl ((5-sulfamoylfuran-2-yl)methyl)carbamate (100 mg, 0.456 mmol) was dissolved in dry THF (3 mL). To this solution, tBuOK (1M in dry THF, 0.5 mL, 0.50 mmol) was added at 0 °C under argon and the reaction mixture was stirred at 0 °C for 5 min. Then 4-isocyanato-1,2,3,5,6,7-hexahydro-*s*-indacene (100 mg, 0.50 mmol) was dissolved in dry THF (2 mL) and added to the reaction mixture at 0 °C. The reaction mixture was stirred for 4 hrs at room temperature under argon atmosphere. The organic solvent was removed under reduced pressure. The crude product was purified by silica column chromatography using chloroform-methanol mixtures as eluent to yield Compound-4 as a white solid (183 mg).

Yield 96%; 1H NMR (600 MHz, DMSO-d6) δ 1.27 (t, J = 7.1 Hz, 3H), 1.91 (q, J = 7.4 Hz, 4H), 2.65 (t, J = 7.4 Hz, 4H), 2.76 (t, J = 7.4 Hz, 4H), 4.24 (q, J = 7.1 Hz, 2H), 6.82 (bs, 1H), 6.88 (bs, 1H, NH), 7.21 (bs, 1H), 7.70 (bs, 1H, NH), 8.39 (bs, 1H, NH). HRMS (ESI) m/z [M-H]+ calculated for C20H21N2O6S 417.1199, found 417.1139.

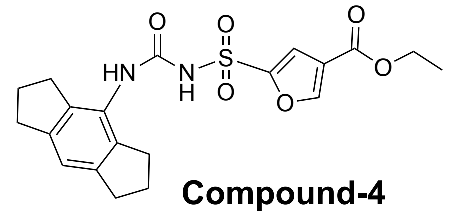

**Compound-4**

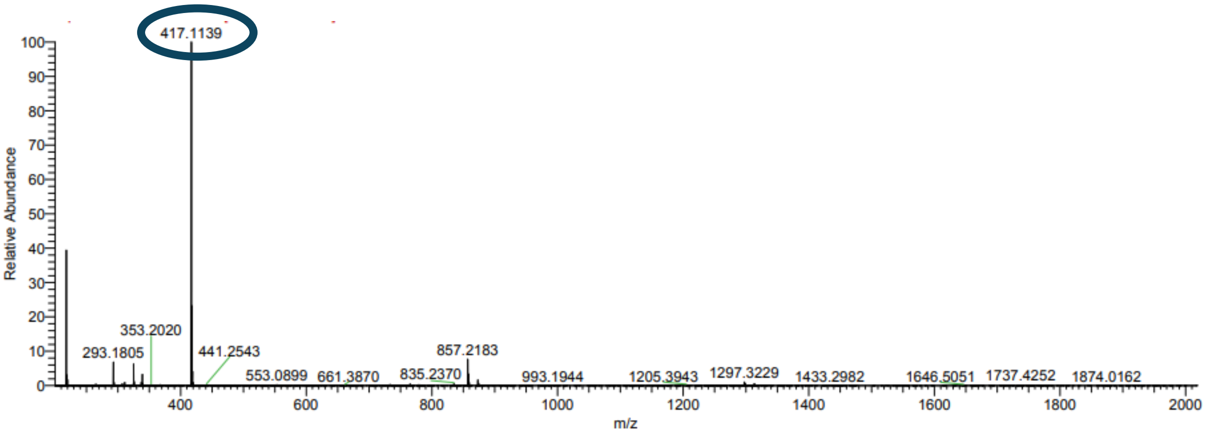


HRMS spectrum of Compound-4.

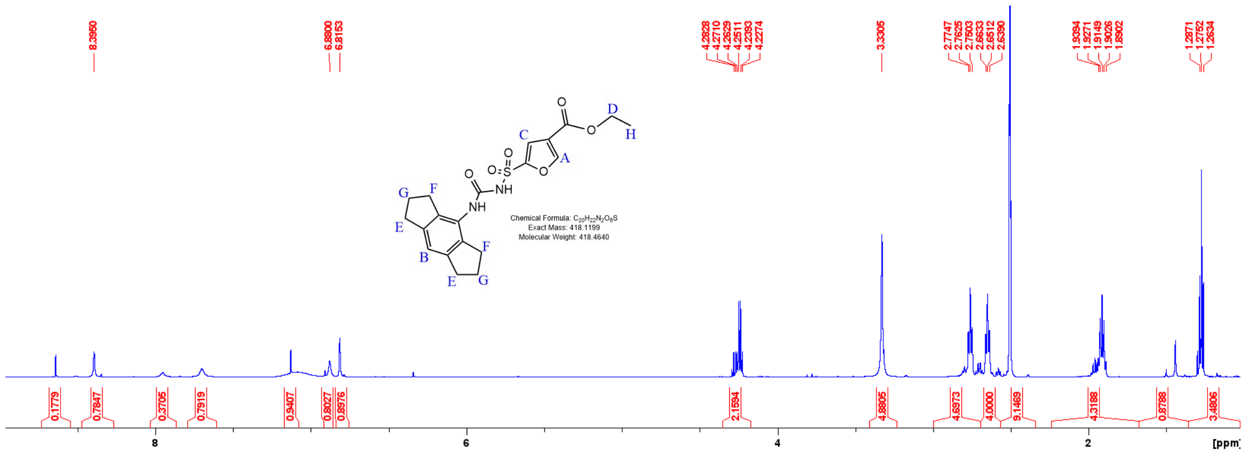


1H-NMR spectrum of Compound-4.

**Synthesis of Compound-1**

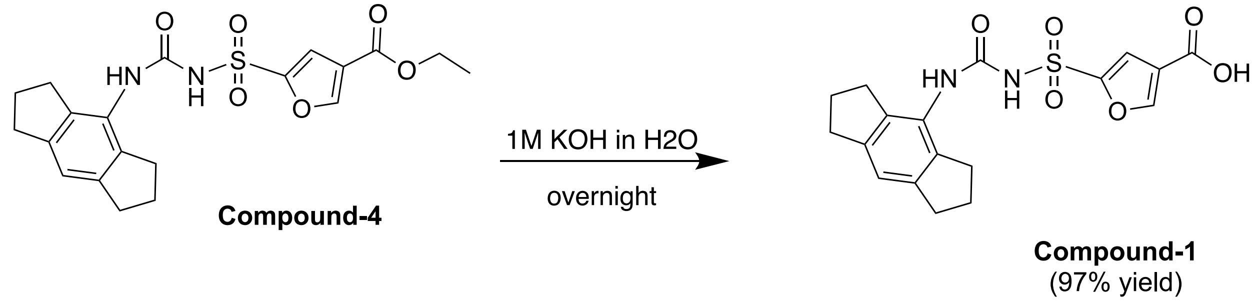

Method: **Compound-4** (183 mg, 0.438 mmol) was dissolved in THF (3 mL). To this solution, KOH (1.0 M in water, 1.5 mL, 1.50 mmol) was added at 0 °C under argon and the reaction mixture was stirred at room temperature for overnight under argon atmosphere. The organic solvent was removed under reduced pressure. The crude product was purified by silica column chromatography using dichloromethane-methanol mixtures as eluent to yield **Compound-1** as a white solid (166 mg).

Yield 97%; 1H NMR (600 MHz, DMSO-d6) δ 1.90 (q, J = 7.4 Hz, 4H), 2.66 (t, J = 7.4 Hz, 4H), 2.76 (t, J = 7.4 Hz, 4H), 6.67 (s, 1H), 6.78 (s, 1H), 7.30 (bs, 1H, NH), 7.59 (bs, 1H, NH), 7.91 (s, 1H). HRMS (ESI), m/z [M-H]+ calculated for C18H17N2O6S 389.0886, found 389.0824.

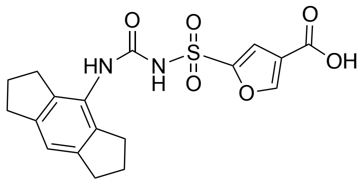


**Compound-1**

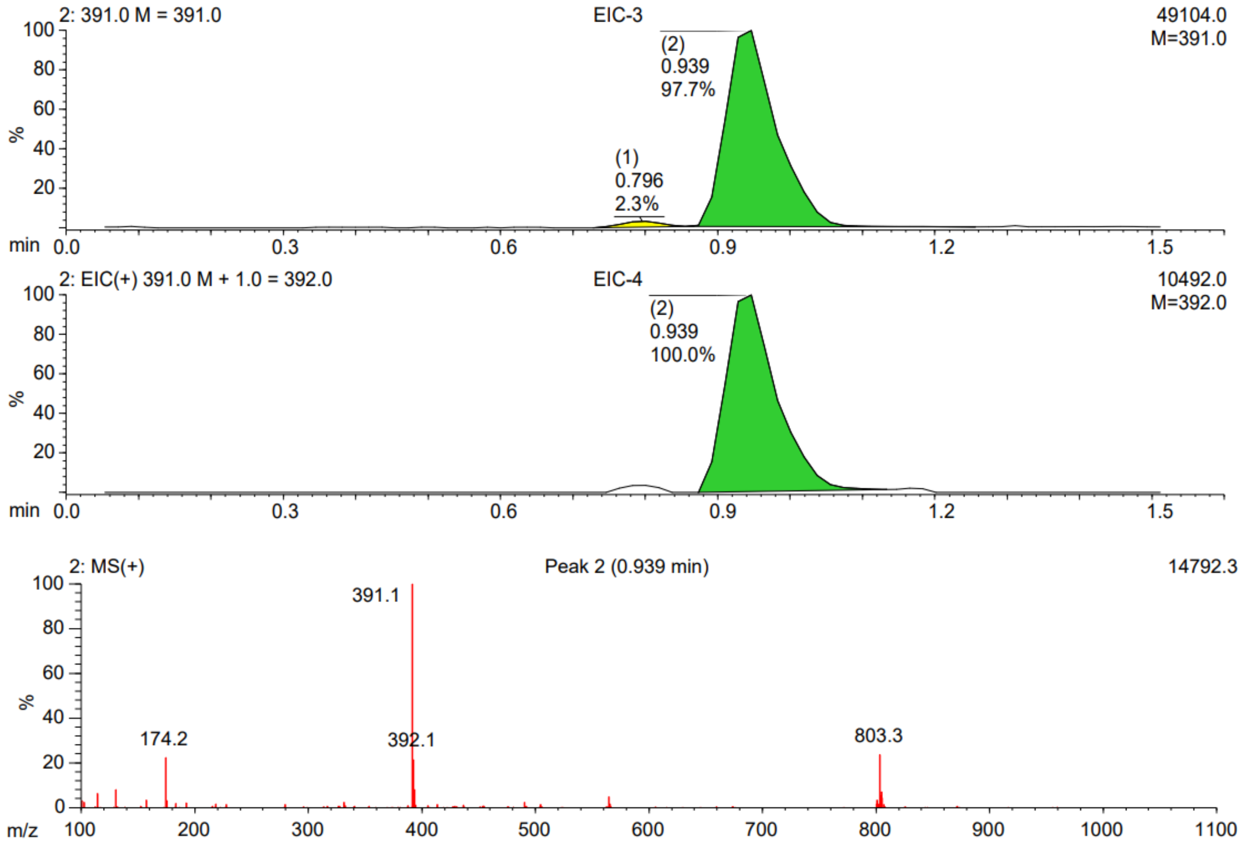


LRMS spectra of Compound-1.

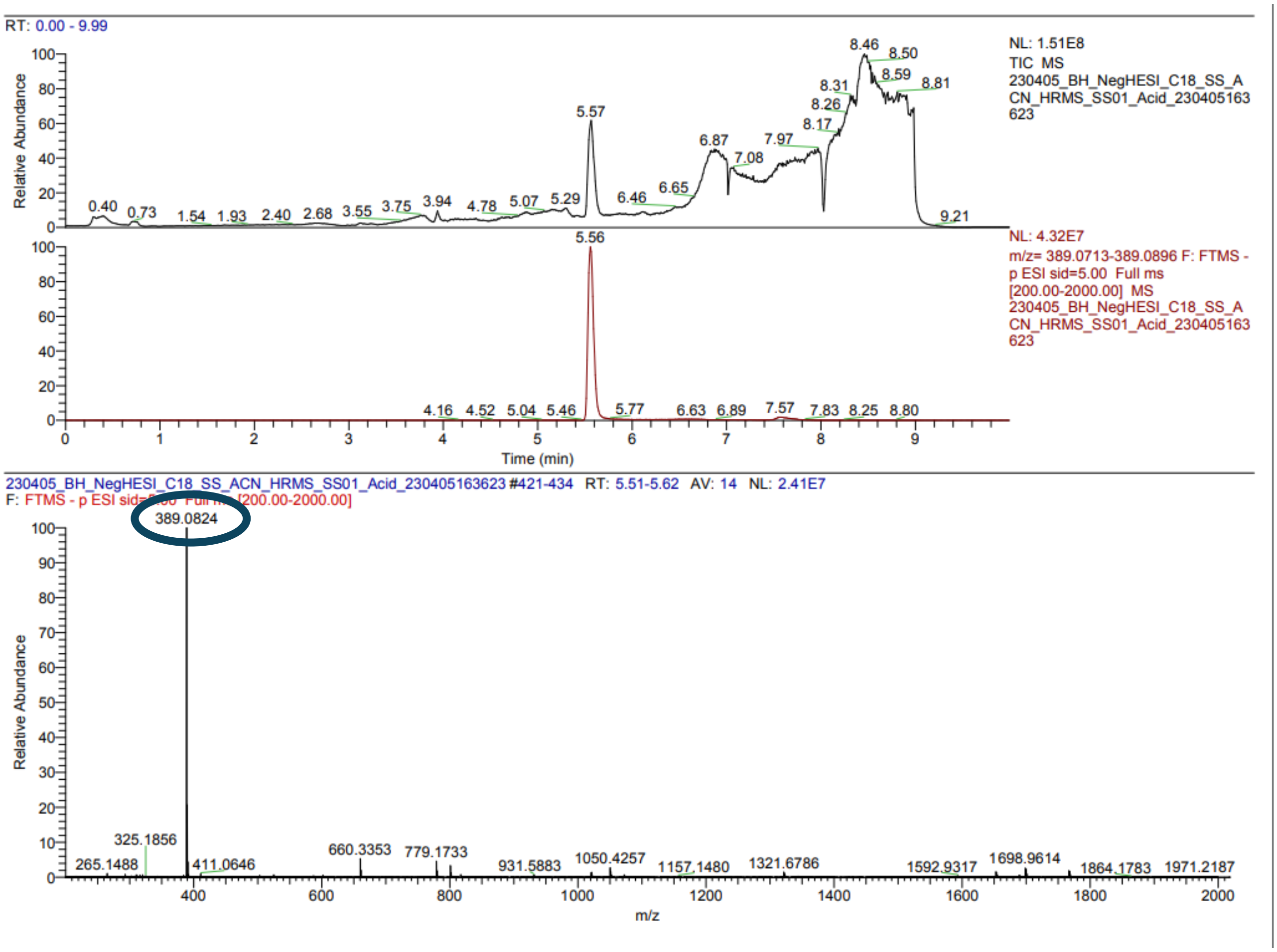


HRMS spectrum of Compound-1.

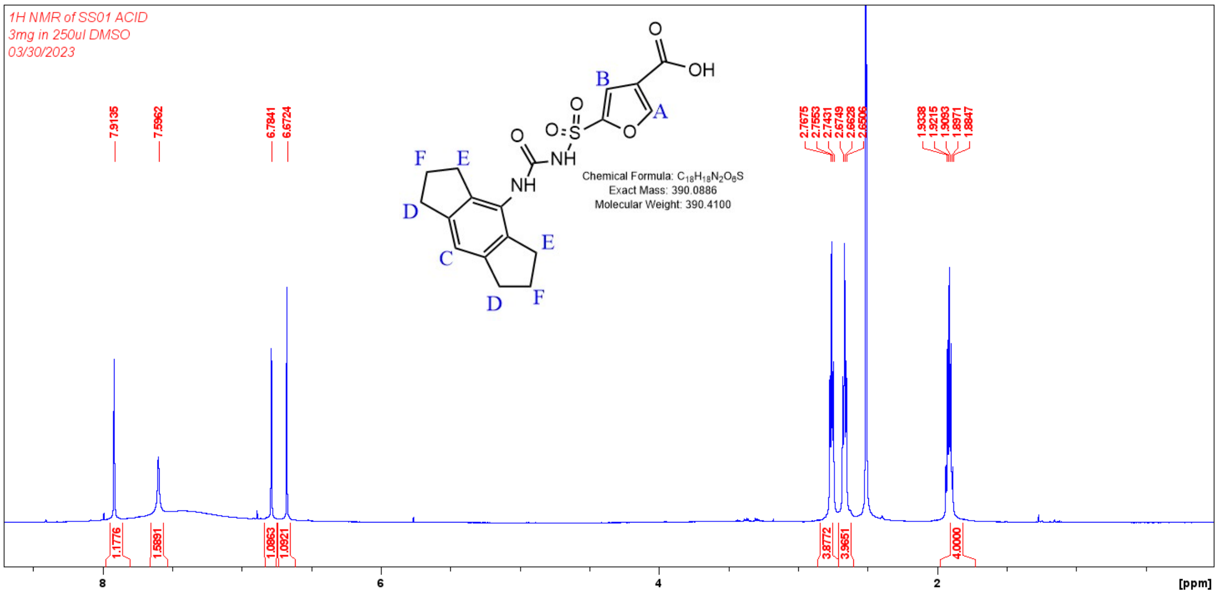


1H-NMR spectrum of Compound-1.

| | | | | |
|---|---|---|---|---|
| | **Table S1: Microscope configuration for TUNEL assay** | | | |
| Microscopy and image processing details pertaining to Figure 4 and Figure 11 | | | | |
| **Image Dimensions** | | | | |
| Scaling (per pixel) | | 0.42 µm x 0.42 µm | | |
| Image size (pixels) | | 212.5 x 212.5 | | |
| Image size (scaled) | | 425.10 µm x 425.10 µm | | |
| Bit depth | | 8 bit | | |
| **Acquisition Information** | | | | |
| Software | | ZEN Black Edition (V2.4, SP1) | | |
| Microscop | Zeiss™ LSM 710, AxioObserver | | | |
| Objective | | Plan-Apochromat 20x/0.8 M27 | | |
| Beam splitter | | MBS 488/405 | | |
| Acquisition mode | | Sequential multichannel acquisition | | |
| | | **DAPI** | | **TUNEL** |
| Excitation wavelength/laser | | 405 nm: 2.0% | | 633 nm: 2.4 % |
| Emission wavelength | | 455 nm | | 647 nm |
| Detector type | | PMT | | PMT |
| Detector gain | | 600 | | 670 |
| **Image Processing** | | | | |
| **Software** | | **Processing steps** | | |
| ZEN Blue edition (V2.6) | | Pseudocolor, scale bar, and merge | | |
| PowerPoint (V2112) | | Brightness: +20% | | |

| | | | | |
|---|---|---|---|---|
| | **Table S2: Microscope configuration for in vitro imaging** | | | |
| | | | | |
| | Microscopy and image processing details pertaining to Figure 5 | | | |
| | | | | |
| **Image Dimensions** | | | | |
| | | | | |
| Scaling (per pixel) | | 0.55 µm x 0.55 µm | | |
| | | | | |
| Image size (pixels) | | 512 x 512 | | |
| | | | | |
| Image size (scaled) | | 283.40 µm x 283.40 µm | | |
| | | | | |
| Bit depth | | 8 bit | | |
| | | | | |
| **Acquisition Information** | | | | |
| | | | | |
| Software | | ZEN Black Edition (V2.4, SP1) | | |
| | | | | |
| Microscop | Zeiss™ LSM 710, AxioObserver | | | |
| | | | | |
| Objective | | Plan-Apochromat 20x/0.8 M27 | | |
| | | | | |
| Beam splitter | | MBS 458/415 | | |
| | | | | |
| Acquisition mode | | Sequential multichannel acquisition | | |
| | | | | |
| | | **DAPI** | | **InflammaProbe-2** |
| | | | | |
| Excitation wavelength/laser | | 405 nm: 10.0% | | 488 nm: 15.0% |
| | | | | |
| Emission wavelength | | 455 nm | | 516 nm |
| | | | | |
| Detector type | | PMT | | PMT |
| | | | | |
| Detector gain | | 600 | | 670 |
| | | | | |
| **Image Processing** | | | | |
| | | | | |
| **Software** | | **Processing steps** | | |
| | | | | |
| ZEN Blue edition (V2.6) | | Pseudocolor, scale bar, and merge | | |
| | | | | |
| PowerPoint (V2112) | | Brightness: +20% | | |
| | | | | |

| Table S3: Microscope configuration for in vitro imaging | | |
|---|---|---|
| Microscopy and image processing details pertaining to Figure 6 | | |
| **Image Dimensions** | | |
| Scaling (per pixel) | 0.83 µm x 0.83 µm | |
| Image size (pixels) | 512 x 512 | |
| Image size (scaled) | 425.10 µm x 425.10 µm | |
| Bit depth | 8 bit | |
| **Acquisition Information** | | |
| Software | ZEN Black Edition (V2.4, SP1) | |
| Microscop | Zeiss™ LSM 710, AxioObserver | |
| Objective | Plan-Neofluar 20x/0.50 M27 | |
| Beam splitter | MBS 458/415 | |
| Acquisition mode | Sequential multichannel acquisition | |
| | **DAPI** | **InflammaProbe-2** |
| Excitation wavelength/laser | 405 nm: 0.9% | 514 nm: 15.0% |
| Emission wavelength | 455 nm | 516 nm |
| Detector type | PMT | PMT |
| Detector gain | 600 | 750 |
| **Image Processing** | | |
| **Software** | **Processing steps** | |
| ZEN Blue edition (V2.6) | Pseudocolor, scale bar, and merge | |
| PowerPoint (V2112) | Brightness: +20% | |

| | | | | |
|---|---|---|---|---|
| | **Table S4: Microscope configuration for ex vivo imaging** | | | |
| | Microscopy and image processing details pertaining to Figure 10 | | | |
| **Image Dimensions** | | | | |
| Scaling (per pixel) | | 0.83 µm x 0.83 µm | | |
| Image size (pixels) | | 512 x 512 | | |
| Image size (scaled) | | 425.10 µm x 425.10 µm | | |
| Bit depth | | 8 bit | | |
| **Acquisition Information** | | | | |
| Software | | ZEN Black Edition (V2.4, SP1) | | |
| Microscop | Zeiss™ LSM 710, AxioObserver | | | |
| Objective | | Plan-Apochromat 20x/0.8 M27 | | |
| 3eam splitter | | MBS 488/461/633 | | |
| Acquisition mode | | Sequential multichannel acquisition | | |
| | | **InflammaProbe-2** | | **NLRP3** |
| Excitation wavelength/laser | | 488 nm: 2.0% | | 633 nm: 4.5 % |
| Emission wavelength | | 516 nm | | 697 nm |
| Detector type | | PMT | | PMT |
| Detector gain | | 613 | | 600 |
| **Image Processing** | | | | |
| **Software** | | **Processing steps** | | |
| ZEN Blue edition (V2.6) | | Pseudocolor, scale bar, and merge | | |
| PowerPoint (V2112) | | Brightness: +20% | | |

**Table S5: Microscope configuration for in vitro imaging**

Microscopy and image processing details pertaining to Figure 7, 8 and Figure S4

| | | | |
|---|---|---|---|
| **Image Dimensions** | | | |
| Scaling (per pixel) | | 0.83 µm x 0.83 µm | |
| Image size (pixels) | | 512 x 512 | |
| Image size (scaled) | | 425.10 µm x 425.10 µm | |
| Bit depth | | 8 bit | |
| **Acquisition Information** | | | |
| Software | | ZEN Black Edition (V2.4, SP1) | |
| Microscope | Zeiss™ LSM 710, AxioObserver | | |
| Objective | | Plan-Apochromat 20x/0.8 M27 | |
| Beam splitter | | MBS 488/405 | |
| Acquisition mode | | Sequential multichannel acquisition | |
| | | **DAPI** | **InflammaProbe** |
| Excitation wavelength/laser | | 405 nm: 10.0% | 488 nm: 15.0% |
| Emission wavelength | | 455 nm | 516 nm |
| Detector type | | PMT | PMT |
| Detector gain | | 600 | 670 |
| **Image Processing** | | | |
| **Software** | | **Processing steps** | |
| ZEN Blue edition (V2.6) | | Pseudocolor, scale bar, and merge | |
| PowerPoint (V2112) | | Brightness: +20% | |
